# Impact of MR Acquisition Parameters on DTI Scalar Indexes: A Tractography Based Approach

**DOI:** 10.1371/journal.pone.0137905

**Published:** 2015-10-12

**Authors:** Gonzalo Barrio-Arranz, Rodrigo de Luis-García, Antonio Tristán-Vega, Marcos Martín-Fernández, Santiago Aja-Fernández

**Affiliations:** Laboratorio de Procesado de Imagen, Departamento de Teoría de la Señal y Comunicaciones e Ingeniería Telemática/ETSI Telecomunicación, Universidad de Valladolid, Valladolid, España; Chinese Academy of Sciences, CHINA

## Abstract

Acquisition parameters play a crucial role in Diffusion Tensor Imaging (DTI), as they have a major impact on the values of scalar measures such as Fractional Anisotropy (FA) or Mean Diffusivity (MD) that are usually the focus of clinical studies based on white matter analysis. This paper presents an analysis on the impact of the variation of several acquisition parameters on these scalar measures with a novel double focus. First, a tractography-based approach is employed, motivated by the significant number of clinical studies that are carried out using this technique. Second, the consequences of simultaneous changes in multiple parameters are analyzed: number of gradient directions, b-value and voxel resolution. Results indicate that the FA is most affected by changes in the number of gradients and voxel resolution, while MD is specially influenced by variations in the b-value. Even if the choice of a tractography algorithm has an effect on the numerical values of the final scalar measures, the evolution of these measures when acquisition parameters are modified is parallel.

## Introduction

Diffusion Magnetic Resonance Imaging (dMRI) is an MRI imaging technique that allows the quantification of the diffusivity of water molecules within the tissue, providing information about the organization of the white matter of the brain and the orientation of its fiber tracts [1]. dMRI has gathered an extraordinary interest among the scientific community during the last years due to the relationships found between a number of neurological and neurosurgical pathologies and alterations in the white matter as revealed by an increasing number of dMRI studies [2–5].

The most popular model of the diffusion profile is based on the Gaussian assumption, which allows the diffusion to be modeled with a single covariance matrix, namely the second order diffusion tensor. This technique is known as Diffusion Tensor Imaging (DTI) and requires measurements at least six different diffusion directions. However, it is today feasible to acquire a larger number of diffusion weighted images in clinical time, allowing more complex models of the diffusion profile usually known as High Angular Resolution Diffusion Imaging (HARDI) [6]. An advantage of HARDI is that it can better model complicated fiber architectures such as crossing, bending, or kissing fibers.

White matter studies using dMRI usually rely on the comparison of scalar measures that quantify the diffusion within a voxel. The most common measures, which are based on the DTI model, are the Fractional Anisotropy (FA), which measures to what extent a diffusion direction is dominant over the others, and the Mean Diffusivity (MD), which quantifies the total amount of diffusion. Other relevant scalar measures include the tensor mode and the linear measure.

Whatever the diffusion model, white matter analysis is commonly performed in clinical studies following a volumetric or a tract-based approach. In the former case, Regions of Interest (ROIs) are either manually or automatically defined, and then the scalar quantities derived from dMRI can be compared across subjects. Alternatively, images can be registered to a common template and then voxelwise statistics are computed in the whole brain or in certain areas of interest (VBM, Volumetric-Based Morphology) [7]. In the latter case, tractography is carried out using the diffusion information in the dMRI in order to achieve a representation of the neural pathways [8, 9]. Finally, measures are derived from the obtained fibers in order to compare corresponding fiber bundles in different subjects.

In spite of the enormous number of studies performed during the last two decades using this technique, dMRI suffers from a major limitation: the scalar values upon which these studies are based are heavily dependent on a number of factors. These include the number of scan repetitions [10], the diffusion sensitivity b-value [11, 12], the number of gradient sampling directions [13], the scheme of these directions [14, 15], the voxel spacing resolution [16], the trade-offs between some of them [17] or even the scanner manufacturer [18]. Other factors that can be a source of intra-session variability are the MR signal variation and subject physiological noise, motion and positioning [10, 15]. All these elements have an influence on the voxel values of the DTI or HARDI volume.

Besides, the white matter analysis method (i.e. volumetric or tract-based) also represents a source of variability. In VBM there has been much debate about its advantages and drawbacks [19–21], the main concern being how it can be guaranteed that the observed changes are a consequence of actual changes in the white matter instead of the effect of an incorrect alignment in the registration process. Tract-based spatial statistics (TBSS), a volumetric analysis restricted to an FA skeleton of the white matter, has been proposed as a more robust alternative and has gained considerable popularity in the last years [21]. With regard to tract-based approaches, several studies have analyzed the reproducibility of different tractography algorithms in either synthetic phantoms and real data [22–24]. The reproducibility of dMRI scalar measures from tractography in real data has also been studied [18, 25–27] showing, using different tractography algorithms, that the intra-subject reproducibility of the scalar measures depends on the white matter structure (i.e. corpus callosum) and the scalar measure considered (i.e. tractography volume, FA, MD). Moreover, the reproducibility and accuracy of these studies is known to be limited by additional factors, such as the tractography algorithm employed and its parameters. Finally, it has been shown that the tractography selection method has an significant impact on the results of white matter analysis [28].

In summary, there is solid evidence in the literature showing that the the potential use of dMRI as a diagnostic tool, as well as the comparison of results from different white matter studies, is limited by the multiple factors influencing the analysis of dMRI data (see Table 1 for a summary of the most relevant findings [10, 12–14, 25, 29–37]).

**Table 1:** Summary of the main studies in the literature devoted to the analysis of different acquisition factors on DTI scalar measures.

Study	Analyzed parameter	Analyzed Measure	Main finding
[12]	b-value (700-1000)	FA	Average FA decreases with increasing b-value.
[29]	b-value (500-2500)	FA, MD, AD, RD	Small FA variation with varying b-value (variation depends on tissue type and age). MD, AD and RD decrease with increasing b-value.
[30]	b-value	FA, MD, AD, RD	FA error due to PVE decreases with higher b-value. MD decreases with increasing b-value. In presence of PVE, FA decreases with higher b-values, while MD, AD and RD increase.
[31]	b-value	FA	In regions with intermediate FA, high b-value (>3000) and low SNR produce FA underestimation and eigenvector shift.
[32]	b-value	FA, MD	Simulated data (FA = 0.9). Correct FA estimation for b-values between 500 and 1500. FA underestimation for b-values over 3000. MD decreases with increasing b-values.
[33]	b-value (160-800)	FA	Phantoms and real data. Increasing b-values reduces standard dev. of FA.
[34]	Resolution	FA	Smaller voxel size increases FA.
[35]	Resolution, gradient directions	FA	Smaller voxel size increases FA. A higher number of gradient directions decreases FA.
[36]	Resolution	MD	MD does not change with voxel size.
[13]	Gradient directions	FA	Simulated data. A higher number of gradient directions decreases estimated FA and its standard deviation.
[14]	Gradient directions, SNR	FA, MD	A higher number of gradient directions decreases estimated FA and its standard deviation. Importance of a well-balanced gradient directions scheme. Higher SNR reduces standard deviation in FA and MD (simulated data).
[25]	Gradient directions, SNR	FA, MD	A higher number of gradient directions increases estimated FA while reducing MD. Lower noise increases FA.
[37]	Gradient directions	FA, MD, RA	20 gradient directions are enough for FA and MD estimation. 30 are needed for RA.
[10]	SNR	FA, MD	Higher noise increases FA, while MD does not change.

Even though many of the factors with an influence on the dMRI data have been studied before, they have usually been analyzed independently. The combined effects of the acquisition parameters on the scalar measures employed in white matter studies has not been thoroughly studied.

This paper, thus, evaluates the influence of the major DTI acquisition parameters (b-value, number of diffusion orientations and voxel spacing resolution) and the choice of a tractography algorithm on the output of a white matter analysis of some major fiber bundles of the brain using a tract-based approach.

As there are many possible combinations of factors that could be studied, we have centered our efforts on those acquisition parameters that are commonly adjusted when performing DTI scans in clinical applications or DTI group studies, on the one hand, and on those DTI-derived scalar measures that have a clear potential as quantitative biomarkers. There exists previous literature that studies the effect of some parameters independent, but the main contribution of this paper is to show the combined effect of these three main acquisition parameters.

Nevertheless, more information about other possible combinations of parameters can be found on the supplementary information that accompanies this paper: the MATLAB data in S2 File and the tables in S3 File.

A glossary of the terms employed on this paper can be seen in Table 2.

**Table 2:** Glossary of employed terms.

FA	Fractional Anisotropy
MD	Mean Diffusivity
AD	Axial Diffusivity
RD	Radial Diffusivity
∣ℱ∣	Number of Fibers
$V_{B}$	Bundle Volume
$\bar{L}$	Track Length
OV	Overlapping Volume
CC	Corpus Callosum
CCA	Corpus Callosum—Anterior
CCG	Corpus Callosum—Genu
CCP	Corpus Callosum—Posterior
CCS	Corpus Callosum—Splenium
CG	Cingulate Gyrus (cingulum)
CGL	Cingulate Gyrus (cingulum)—Left
CGR	Cingulate Gyrus (cingulum)—Right
RKT	Runge Kutta Tractography
GT	Global Tractography
SNR	Signal to Noise Ratio
ROI	Region Of Interest
PVE	Partial Volume Effect
MRI	Magnetic Resonance Imaging
DWI	Diffusion Weighted Imaging
DTI	Diffusion Tensor Imaging
WLS	Weighted Least Squares
TE	Echo Time
TR	Repetition Time
AC-PC	Anterior Commisure—Posterior Commisure
SE-EPI	Spin-Echo Echo-Planar Image

## Materials and Methods

The main objective of this paper is the quantification of the variability of the scalar measures most commonly employed in clinical studies as a function of the acquisition parameters. To that end, data sets from 13 different control subjects obtained with different combinations of the acquisition parameters are considered. Scalar measures were derived from two major white matter fiber bundles (Corpus Callosum and Cingulum) from the results of two different tractography algorithms: a streamline Runge-Kutta tractography (RKT) algorithm and a global tractography (GT) algorithm [23].

Four DTI scalar measures were considered: Fractional Anisotropy (FA), Mean Diffusivity (MD), Axial Diffusivity (AD) and Radial Diffusivity (RD), as defined in [38]. Additionally, four fiber-related measures were also analyzed: number of fibers obtained by the tractography algorithm, average length of the fiber tracts in each bundle, total volume occupied by the tracts of each bundle and overlapping volume between reconstructions.

### Subjects and data acquisition

Thirteen healthy male adults, aged between 23 and 31 (average age 27 years), participated in this study. All volunteers signed an informed consent and the study was approved by the Ethics Comitee of the Gregorio Marañón Hospital (Madrid). All subjects had normal neurological scans and no history of neurological diseases. Datasets were acquired in a PHILIPS 1.5 T MR scanner at the Gregorio Marañón Hospital.

Diffusion weighted MR images were acquired using a multi-shot pseudo-3D double spin-echo echo-planar imaging (SE-EPI) sequence. Each exam was composed of nine different DTI acquisitions with different combination of parameters, resulting in a total of 117 scans. The scanning parameters employed were:

Diffusion sensitivity b-value: Three different b-values were used: 800, 1000 and 1300 *s*/*mm* ^2^. A b-value of 1000 *s*/*mm* ^2^ is the typical value employed on most of clinical DW Imaging applications of the brain for adults [39]. For each one, a reference image with no diffusion weighted (0 *s*/*mm* ^2^) was also acquired. The top b-value of 1300*s*/*mm* ^2^ was selected as a value which is sometimes used in a clinical setting, while still able to obtain an acceptable Signal To Noise Ratio (SNR). The SNR of the baseline does not change for the different b-values, although the SNR of the DWI signal exhibits a small downward trend as the b-value increases.
Spacing Resolution: Scans were acquired with three different voxel sizes: 2 × 2 × 2 *mm* ^3^ (high resolution), 2.5 × 2.5 × 2.5 *mm* ^3^ (medium resolution) and 3 × 3 × 3 *mm* ^3^ (low resolution). Matrix sizes varied between 80 × 80 × 44 and 128 × 128 × 62 voxels, with a Field of View between 240 × 240 *mm* and 256 × 256 *mm*. A total of 66, 53 and 44 sections, respectively, covered the entire brain without gaps.
Gradient directions: All the scans were acquired with 61 gradient directions and one baseline volume. The gradient directions were specifically acquired so that they can be subsampled to 40, 21 or 6 gradient directions while remaining equally spaced for each configuration. This subsampling technique allows the measurement of the effect of different number of gradients with only one data acquisition. According to Vos et al. [40], we can reduce the variance on DTI indexes due to noise to a minimum by using a number of gradients of 40 or higher. We have chosen this configuration of equiseparated directions to highlight the effect of variance due to noise; we have specifically selected 6 gradients plus the baseline as the minimal configuration possible [41].

A survey of these parameters is on Table 3. Other diffusion acquisition parameters are: echo time (TE) 1.6 *ms*, repetition time (TR) 8 *ms*. Slices were aligned parallel to the AC-PC line.

**Table 3:** Parameter set employed on the experiments.

R (voxel Resolution)	2, 2.5, 3 mm^3^
B (b-value)	800, 1000, 1300 s/mm^2^
G (number of Gradients)	6, 21, 40, 61
Tractography algorithms	Runge Kutta, Global

### Tensor Estimation

The diffusion tensor model is employed to characterize the diffusion at each voxel. It assumes that the diffusion profile can be modeled as a 3D Gaussian distribution with three orthogonal eigenvectors or axes of diffusion (the major, median and minor eigenvector $\vec{e_{1}}$,$\vec{e_{2}}$ and $\vec{e_{3}}$ and their corresponding eigenvalues *λ*_1_, *λ*_2_ and *λ*_3_). The six independent elements of the diffusion tensor were estimated using a Weighted Least Squares approach (WLS) [42] for each voxel.

### Tractography Algorithms

Whole brain tractography was performed employing two different tractography algorithms, both using the DTITool software developed by M. Reisert and V. Kiselev at the University of Freiburg [43].

Streamline tractography algorithm (RKT): Starting from a seed point position, this method uses a fourth order Runge Kutta numeric integrator to propagate the tracts both in the positive and negative direction of the local major eigenvector. Seeds were placed at voxels with a FA value over 0.3. The maximum angle allowed between two consecutive steps was 40°, and fibers were discarded if they crossed only five voxels or less. Four fibers were initiated for every seed voxel, starting at random positions within it. Tractography stopped at FA values lower than 0.01, in order to obtain the longest possible track length. Manual ROI filtering ensures that the number of false positive tracks is kept to a minimum.
Global tractography algorithm (GT): Fiber tracking is formulated in terms of the minimization of an energy terms composed of an internal term that captures the geometric properties of line segments and an external term that captures the similarity of the model to the data. Algorithm parameters are the starting and stopping temperatures (controlling the probability of a particle to break its connection to form another), set to 0.5 and 0.001, respectively, the particle width (*w*_*i*_ = 1.5) and length (*l* = 4.5), which control the maximum fiber curvature, and the weight (*w* = 0.19), which controls the expected number of segments. The algorithm was iterated 5 × 10^7^ times during 50 steps for each data set. The probabilities employed for each iteration were: *p*_*birth*_ = 0.25, *p*_*death*_ = 0.05, *p*_*shift*_ = 0.15, *p*_*opt*_ = 0.1, *p*_*fiber*_ = 0.45. Fibers with lengths under 10 *mm* or over 150 *mm* were discarded.

**Fig 1:**
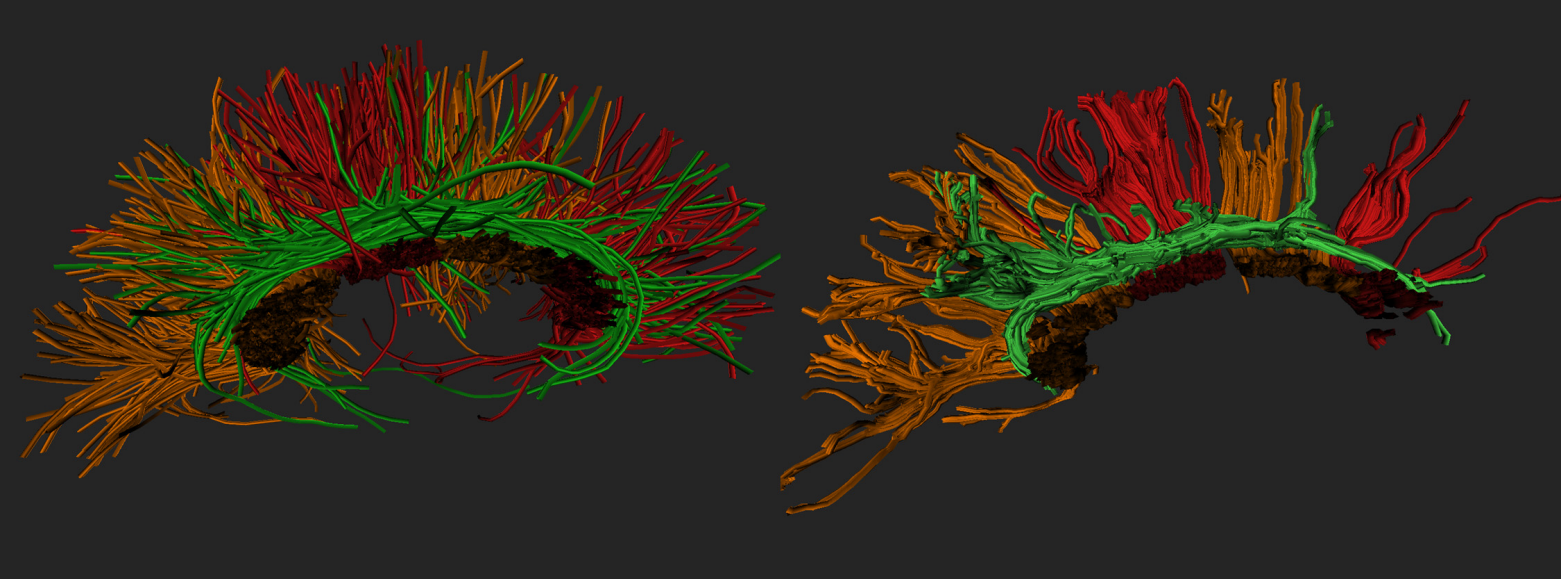
RKT (left) and GT (right) reconstructions of one side of the brain (CC, red and orange; CG, green) for *G* = 61 gradients, *R* = 2 × 2 × 2 *mm* ^3^, *b* = 1000 *s*/*mm* ^2^.

The two tractography algorithms employed were chosen as representative of two major fiber tracking philosophies. Sample reconstructions of the CC and CG fiber bundles using both methods are depicted on Fig 1.

### Tractography Selection

Comparisons were performed on two specific fiber bundles through tractography selection. This procedure was performed using a region of interest (ROI) spatial filtering approach, selecting fibers that travel through at least two ROIs. ROIs were manually placed on FA maps according to the criteria defined in [25]. Based on these criteria, the following fiber bundles were considered for this study: genu of the corpus callosum (CCG); anterior and posterior corpus callosum (CCA and CCP); splenium of the corpus callosum (CCS); left and right cingulate gyrus (CGL and CGR). For the sake of concision and due to space requirements, only results for the CCA and CGL are shown in the paper. Results for all studied bundles can be seen in the Supporting Information Files.

### Scalar Measures

FA, MD, AD, and RD maps were derived from the tensor volumes. From these scalar maps, the following measures were considered:

- The average value and standard deviation of the FA, MD, AD, RD at each fiber bundle, calculated as:
  $$\begin{array}{c} \bar{\text{FA}}=\frac{1}{N}\sum_{i}{\text{FA}}_{i} \end{array}$$ (1)
  $$\begin{array}{c} \sigma_{\text{FA}}=\sqrt{\frac{1}{N}\sum_{i}{\left({\text{FA}}_{i}-\bar{\text{FA}}\right)}^{2}} \end{array}$$ (2)
  where *N* is the number of fiber tracts of each bundle, *i* is the index of each fiber and FA_*i*_ is the average value of FA along the points of each fiber. Eqs (1) and (2) are also valid for the other scalar measures, replacing FA by MD, AD or RD. Eight scalar values were derived for every bundle following the described procedure.
- The number of fibers within a particular bundle, ∣ℱ∣ [25].
- Average length of the fiber tracts in each bundle (in *mm*), $\bar{L}$ [25].
  $$\begin{array}{c} \bar{L}=\frac{1}{N}\sum_{i}L_{i} \end{array}$$ (3)
  where *N* is the number of fiber tracts in each bundle and $L_{i}$ is the length of the *i*-th track.
- The total volume of each fiber bundle, $V_{B}$. It has been suggested that $V_{B}$ can detect anatomical abnormalities due to a pathological condition [27]. It has also been shown, however, that this quantity can be affected by the number of gradients [22, 25, 26]. It is measured as:
  $$\begin{array}{c} V_{B}=N\times \left|V\right| \end{array}$$ (4)
  where ∣*V*∣ is the voxel size of the data volume and N is the number of voxels occupied by the fibers in the bundle under consideration.
- Overlapping volume (OV) between reconstructions [22, 26]. We assume the reconstruction with 61 gradient directions as the golden standard. Then, for each fiber bundle we can define
  $$\begin{array}{c} \text{OV}=N_{61}\cap N_{g} \end{array}$$ (5)
  where $N_{61}$ is the number of voxels occupied by the fibers derived from the tractography algorithm when 61 gradient directions are used and $N_{g}$ is the number of voxels occupied by the fibers in a reconstruction when *g* gradients are employed.

## Results

Since we have resorted in this paper to a tractography-based white matter analysis method, we first provide some results on the robustness of the employed tractography algorithms with respect to changes in the acquisition parameters. Variations in the tractography output due to changes in the acquisition parameters will have a direct influence on the final scalar measures that are eventually derived, and different tractography approaches can perform differently.

**Fig 2:**
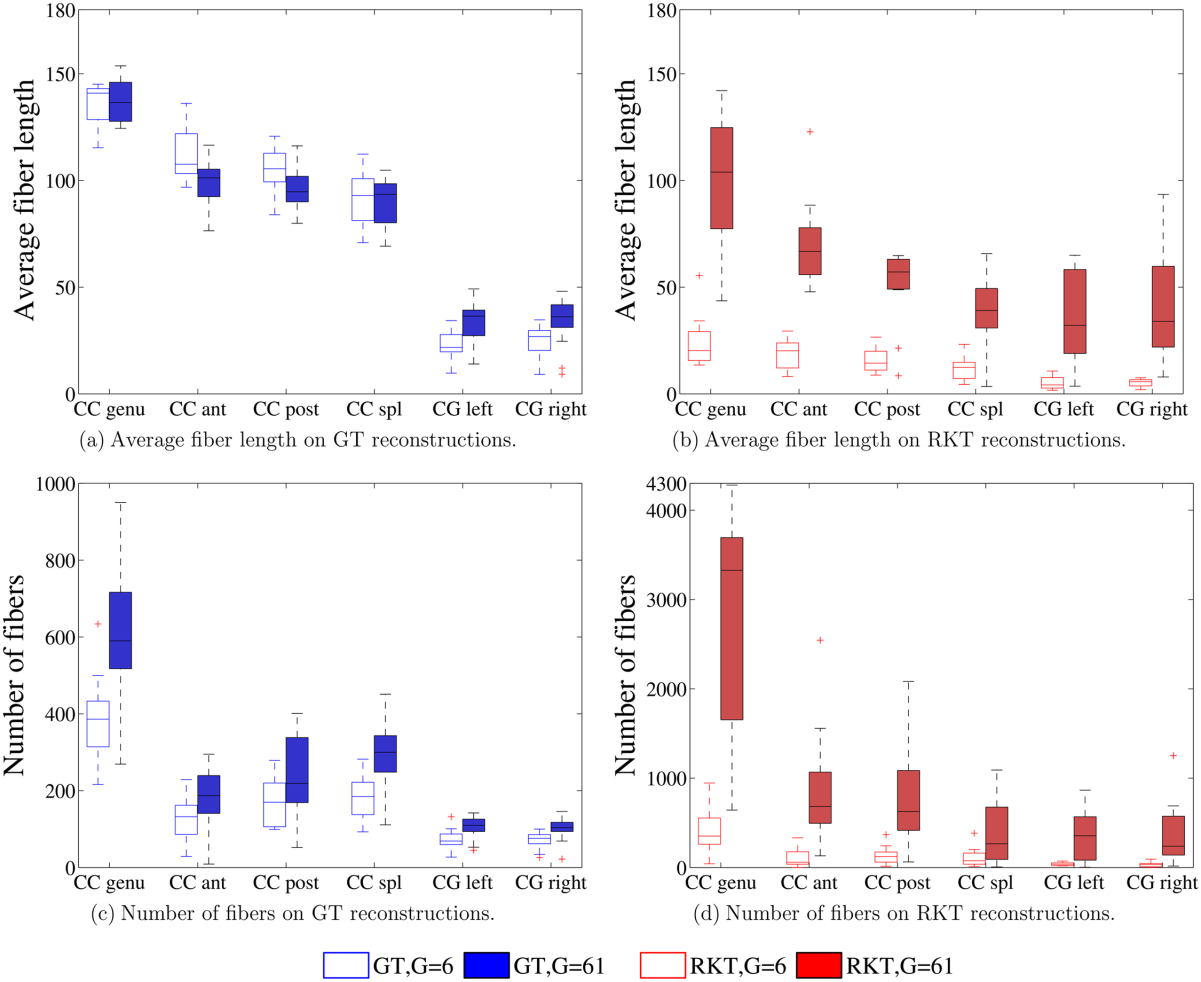
Average fiber length $\bar{L}$ and number of fibers ∣ℱ∣ for the RKT (red) and the GT (blue) reconstructions for *r* = 2*mm* ^3^ and *b* = 1000 *s*/*mm* ^2^. White boxes stand for *G* = 6 gradient directions, while solid boxes stand for *G* = 61. Six different bundles are considered.

Fig 2 shows a comparison of the average fiber length and number of fibers obtained by the GT and RKT tractography methods when considering a configuration with a small number of gradient directions (*G* = 6) and a very high one (*G* = 61). Results indicate RKT to produce a much higher number of fibers than GT. Also, configurations with more gradient directions produce more fibers for both tractography methods. With regard to the average fiber length, GT shows to be relatively insensitive to the number of gradient directions, while RKT is heavily dependent and produces very short fibers when a reduced number of gradient directions is applied.

**Fig 3:**
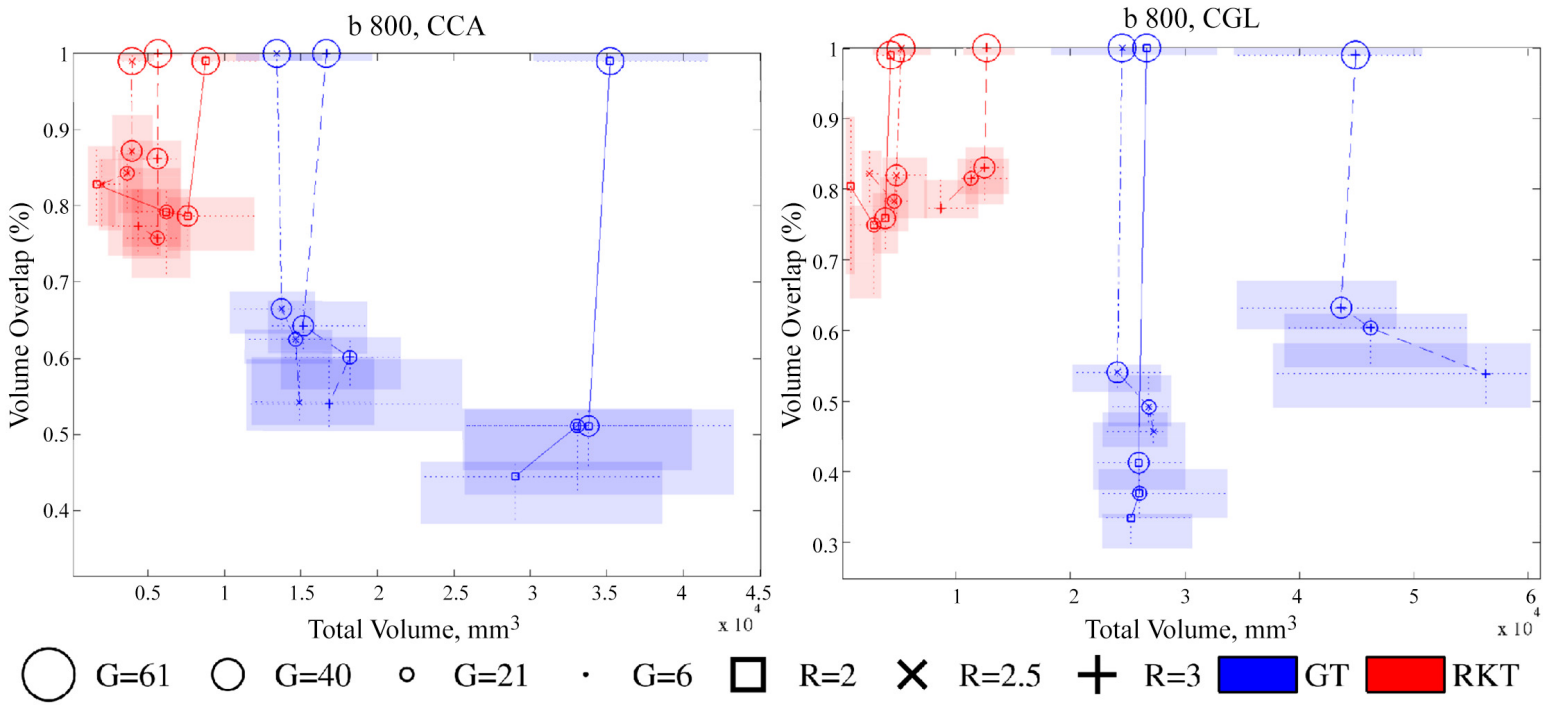
2-D boxplot of Total Volume, $V_{B}$, (*mm* ^3^) versus OV (percentage) for RKT (red) and GT (blue) on CCA and CGL fiber bundles at *B* = 800 *s*/*mm* ^2^, and different configurations of *R* and *G*. The limits of each rectangle indicate the first and third quartile values for both boxplots. Dotted lines denote median values of each boxplot, while solid and dashed lines gather reconstructions that share the same voxel resolution.

Similarly, Fig 3 shows the effect of variations in resolution and number of gradient directions on the total volume ($V_{B}$) and volume overlap (OV) of the tractography results for CCA and CGL. A 2D boxplot combines the graphical representation of two different boxplots on each axis. Colors, markers and lines are used to brand all the different groups. As results indicate, GT consistently produces fiber bundles covering a bigger volume than RKT. However, the volume of the obtained fiber bundle is heavily dependent on the number of gradients when GT is employed, while the effect of this parameter on RKT volume is milder. Finally, the effect of the voxel resolution on the tractography volume is not so clear, as different fiber bundles (CCA and CGL) behave differently although both tractography algorithms show the same evolution in tractography volume when resolution is changed.

Figs 2 and 3 show relevant differences in the output of the two tractography methods that have been considered in this work. These differences will have an effect on the final scalar measures that are usually employed in clinical studies, as will be shown in the remainder of this section and discussed in Section 4.

**Fig 4:**
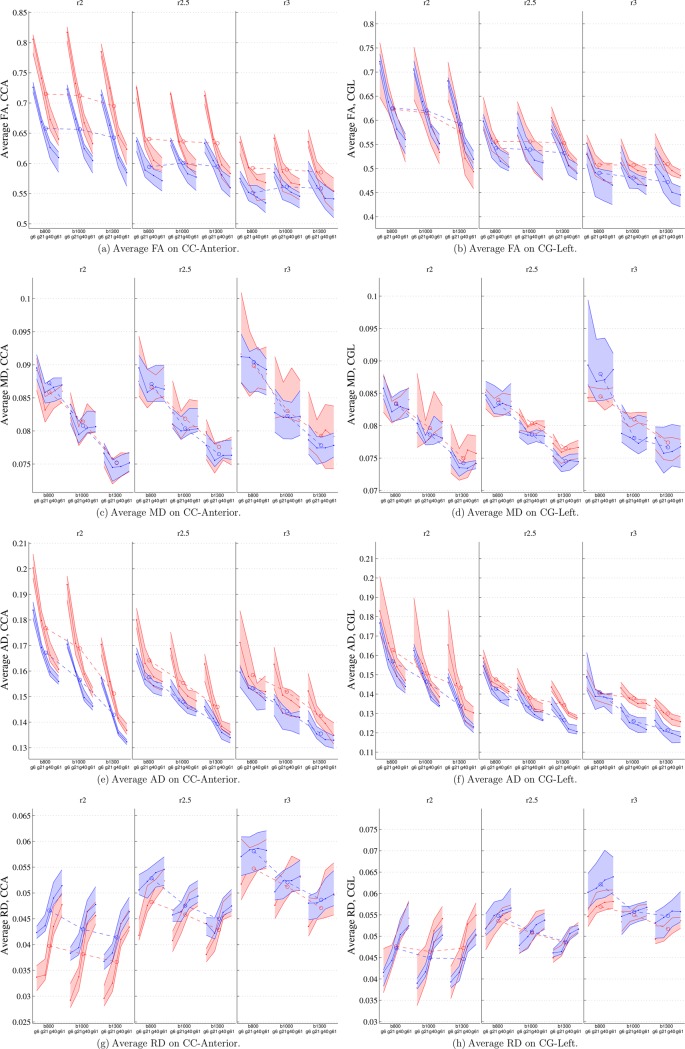
Average values for RKT(red) and GT(blue) on CCA(left column) and CGL (right column) for FA, MD, AD and RD. Central lines represent median values; upper and lower lines of the shaded areas represent the first and third quartile of the data. Dashed lines between groups represent average values of each group.

Next, in order to show the influence of the acquisition parameters on the scalar measures derived from DTI, we show in Fig 4 the average FA, MD, AD and RD along two different fiber bundles using RKT (red) and GT (blue) tractography algorithms, for all different combinations of the acquisition parameters (resolution, b-value and gradient directions). Results for the other fiber bundles considered in this study were consistent with those shown in Fig 4, and were not included for the sake of space.

Results indicate that the number of gradient directions is the acquisition parameter with a higher impact on the different scalar measures, and therefore it is the parameter that most affects the DTI reproducibility. Using both tractography methods, an increase in the number of directions results in an decrease in the average FA and AD, and an increase in the RD. The MD, however, does not follow any clear trend with respect to the number of gradient directions.

Voxel resolution also poses a notable influence on the scalar measures. As seen in Fig 4, a bigger voxel size results in a decrease in the average FA, MD and AD, while increasing the average RD. Interestingly, a bigger voxel size also reduces the variability in FA, AD and RD values due to changes in the number of gradient directions.

Unlike the number of gradient directions or the voxel resolution, the effect of the b-value on some of the scalar measures is not so clear. Variations in the average caused by b-values are shown with a dashed line in Fig 4. The choice of b-value does not seem to pose any clear influence on the average FA values, while the effect on the other scalar parameters is clearer. Higher b-values result in lower MD, AD and, to a lower extent, RD average values.

Finally, the choice of the tractography algorithm can also affect in some cases the average scalar values. For the CC, for instance, the average FA using RKT is higher than that obtained employing GT, while the average RD is lower (specially for small voxel sizes).

**Fig 5:**
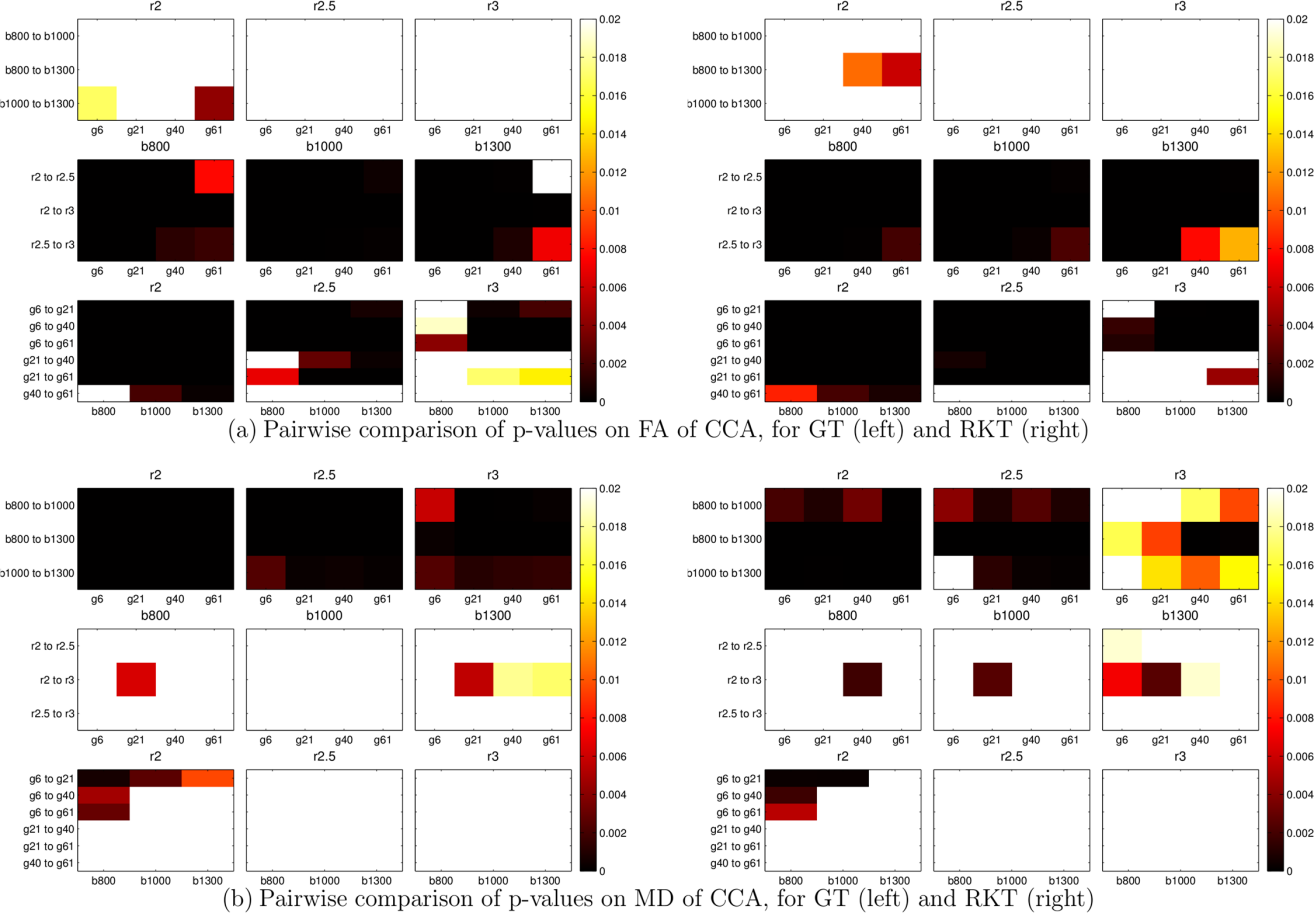
Pairwise comparison of p-values. Black values indicate the existence of a statistical significative difference (*p* < 0.01) between each pair of acq. parameters.

In order to establish the statistical significance of the differences in scalar measures due to acquisition parameters, pairwise t-tests were performed on the average values of FA and MD. Fig 5 shows a graphical representation of the results (statistical significance was considered for *p* < 0.01, and is depicted with orange or darker colors in Fig 5). Results on statistical significance were consistent for both tractography methods. When FA is considered, changes in the number of gradient directions and voxel resolution yield significant changes in the scalar measure (two lower rows), while changes in the b-value do not affect the FA, in general, in a significant way. With regard to the MD, the effect is quite the opposite, as the statistically significant differences arise precisely due changes in b-values (top row), but not due to changes in voxel resolution or the number of gradients.

Table 4 summarizes the influence of the different acquisition parameters considered on the scalar measures and tractography results.

**Table 4:** Summary of the effects of changes of acquisition parameters: b-value (800 to 1300 *s*/*mm* ^2^), number of gradients (6 to 61) and voxel resolution (low Res.-3- to high Res.-2*mm* ^3^-). *μ* stands for average value, *σ* stands for standard deviation.

	↑ Δ*B*	↑ Δ*R*	↑ Δ*G*
FA	↓ *μ* (at HighRes)	↑ *μ*	↓ ↓ *μ*
MD	↓ ↓ *μ*		
		↓ ↓ *σ*	
AD	↓ *μ*	↑ *μ* (spec. on *G* = 6)	↓ ↓ *μ*
	↓ *σ*	↓ *σ*	↓ *σ*
RD	↓ *μ*	↑ ↑ *μ* (spec. on *G* = 6)	↑ ↑ *μ* (spec. at LowRes)
	↓ *σ* (spec. at LowRes)		
Length		↑ ↑ *μ* (GT)	↑ ↑ *μ* (RKT)
		↓ *σ* (RKT) (spec. on *G* = 6)	↑ ↑ *σ* (RKT)
Volume		↓ ↓ *μ* (GT), ↓ *μ* (RKT)	
Number of fibers		↑ *μ* (GT), ↑ ↑ *μ* (RKT)	↑ ↑ ↑ *μ* (RKT)
		↑ *σ* (GT), ↑ ↑ *σ* (RKT)	↑ ↑ ↑ *σ* (RKT)

## Discussion

DTI studies and, particularly, those using tractography-based approaches, suffer from two main concerns: (1) the lack of a golden standard for tractography validation and (2) the problem of reproducibility. It is today well known that the values of scalar measures employed in DTI studies (FA, MD…) are greatly dependent on the acquisition parameters, and on the scanner itself. Most clinical studies using DTI include comparisons between subjects with pathologies and control subjects, all of them scanned in the same machine and with the same acquisition parameters. Multicenter studies, however, remain impractical due to the difficulty of replicating the exact same acquisition protocol. Comparisons between different studies are also hindered by the dependencies of the scalar measures on the acquisition process, and usually only qualitative trends can be compared between studies.

As previous literature advises caution on the use of geometrical measures as potential biomarkers [16], we have decided to focus our analysis on traditional DTI scalar indices.

Choosing an unbiased and robust methodology is of paramount importance when combining results from different datasets. In previous work, authors have chosen different registration methods (baseline registration, FA registration), methods based on an FA skeleton and even random sampling of the whole brain, looking for the highest FA points. For our study, we have resorted to a tractography-oriented scheme in order to provide results that can help gain insight on the behavior of tract-based white matter analysis, a technique which a growing number of works in the literature is based upon. Comparisons are performed for each fiber bundle of interest, regardless of the position of that bundle in each particular subject. However, tractography algorithms are also known to be a possible source of errors and artifacts. To take into account those errors into the study, two different methods, based on completely different tracking philosophies were used.

Results of the experiments carried out in this paper are strongly consistent with the common intuition in the DTI community, and are aligned with most of the studies previously published. Besides showing that the most common scalar measures are greatly influenced by acquisition parameters, they also highlight some important issues to be taken into account when performing tractography studies.

The **number of gradient directions** is the first key acquisition parameter with an influence on the DTI scalar measures. The number of directions is related to the accuracy in the estimation of the diffusion tensor, with more directions meaning a more accurate estimate. However, as previously reported in several works, although the variance of the estimation error in the DT decreases when more gradients are considered, the error bias remains unaltered [42]. The bias of the error is related to the SNR, not to the number of directions. Accordingly, there is a limit in the accuracy we can obtain by increasing the number of gradients. It will also be necessary to increase the SNR, by using multiple repetitions, for instance, to reduce the global error. This effect has been also described in [44] where authors showed that the estimation of the FA is more accurate when the number of gradients grows in number. Again, there is an upper bound in this improvement, as the bias cannot be removed by adding more directions. All in all, datasets with a higher number of directions will correspond to the most accurate estimation.

Accordingly, the effect of the number of directions over the tensor estimation will mainly be reflected on those measures with a directional nature, such as FA, AD and RD. This is precisely the case in the experiments shown in the previous section. According to them, a larger number of gradients goes along with a reduction on the values of FA, a reduction that is more noticeable for smaller voxel sizes. This effect over the FA values has been also reported by [13, 14, 35, 45]. Curiously, Wang et al. described the opposite effect [25].

The shape of the diffusion tensor, and consequently the values of all tensor-derived diffusion measurements, may be otherwise sensitive to the number of acquired gradients due to an additional issue: for the common range of image resolutions, each voxel will comprise not only one coherent group of fiber bundles, but on the opposite it has been estimated that two thirds of the voxels in the white matter will comprise crossing, bending, or kissing fiber bundles in divergent directions. As long as the Gaussian diffusion model does not apply to any of these situations, the diffusion tensor will give rise to a suboptimal data fitting, which will be moreover dependent on the sampling scheme. It seems reasonable expecting that heavily increasing the number of gradients will palliate this issue: if we reach a point for which the spherical sampling is dense enough, it will capture the whole shape of the diffusion pattern, so that the diffusion tensor will represent the best Gaussian fitting of the non-Gaussian data. Once this point is reached, the potential changes in the diffusion measurements due to the number of gradients will be mainly noise-driven.

According to our previous discussion, as well as several previous theoretical and practical works about tensor and FA estimation, the most accurate FA values are those with the higher number of gradient directions (assuming the same number of repetitions per slice and same voxel resolution). Smaller configurations overestimate the values of FA, a fact that needs to be kept in mind when performing clinical research. Note, for instance, that in the CCA (r = 2, b = 1000) values of the FA go from 0.6 to 0.8 when changing the number of directions. This huge range of values is much wider than the usual range between controls and patients in most experiments. For the sake of illustration, see the study in [46], where the average FA in a particular cluster for a control group is 0.4120 vs. 0.3749 for the group under study. The error committed when estimating the FA can indeed be greater than the differences between groups, although by employing the same acquisition parameters for all subjects in a particular study the effect of this error can be removed. The same conclusions can be raised from the results for AD and RD.

Although not specifically analyzed in this paper, a change in the SNR will also affect the FA (and RD and AD). If the SNR increases, the error in the estimation of the diffusion tensor will have a reduced bias. A theoretical analysis in [47] demonstrated that, at low SNR, there is an overestimation of *λ*_1_ and an underestimation of *λ*_3_. AD, RD and indirectly FA, are susceptible to this eigenvalue bias. Note that, sometimes, the variability on the FA, AD and RD when the number of gradient changes is explained as a variation of the SNR, while it is not properly so. The respective contributions of the number of gradients and the SNR over the tensor estimation must not be confused.

On the other hand, the average MD shows to be robust to changes in the number of gradients, with the exception of the behavior for a small number of gradients with low resolution and *b* = 800. This robustness is mainly due to the fact that the choice of more directions will produce a finer estimation of the orientation of the tensor. Thus, oriented magnitudes, such as FA and RD, will be more influenced than MD, which is basically an average value of the magnitude of the tensor. MD will be strongly related to changes in the SNR, however. For a very small number of gradients, there can be an effective reduction of the SNR that can affect the MD, as reported in [14].

As can also be expected, the scalar measures also show significant differences when different **voxel resolutions** are considered. These differences can be explained by a double effect: on the one hand, the SNR of the lower resolution voxels will be higher, so the estimation is more accurate and the bias of the estimation will be smaller. On the other, those low resolution voxels will have a higher partial volume effect (PVE), that is, more underlying tissues are present inside one single voxel. Therefore, the data employed to estimate the diffusion tensor can be corrupted with data from other tissues, which can conversely bias the results. In addition, even when the data inside a single voxel belong to a single tissue, the larger the voxel size, the larger the amount of fiber directions averaged to obtain the scalar measures. The direct result of this effect will be a decrease on the FA value when the voxel size grows. As a consequence of both factors, SNR and PVE, results show a combination of their effects, indicating an increase of the FA at higher resolutions (AD and RD show a similar behavior). This behavior has also been reported by some authors, like [34].

The effect of the resolution on the MD is clearer. For high resolutions there is a decrease in the MD value. Again, this can be explained by the double effect of the PVE and the decrease of the SNR. The same effect is present in the simulations performed in [30].

All in all, variations due to changes in voxel resolution can be explained by changes in the SNR and by PVE. Any bias caused by the latter is hard to quantify, and it will be strongly dependent on the area of interest. Due to the effect of PVE, results opposite to the ones shown here can be obtained when changing the resolution, as different regions can have very different behaviors. Santarelli et al., for instance, reported a decrease of FA at higher resolutions at the cervical spine [35], while Fujiwara et al. reported no significant changes on average MD [34].

The third acquisition parameter analyzed in this work is the **b-value**, which shows a clear effect on the average MD, AD and RD. There is a decrease on the value of MD and AD, and an increase on RD (almost linear) between *B* = 800 and *B* = 1300 *s*/*mm* ^2^. This effect has been previously reported by several authors [29, 30, 32, 45], and is related to the assumptions of the diffusion tensor modeling. Note that MD, AD and RD are diffusion-dispersion related measures. This dependency on the b-value is attributable, according to [45], to the inadequacy of the Gaussian Model to describe the real diffusion process. If the MD measured only the average square displacement per unit time of water molecules, the b-value employed should be irrelevant. However, the diffusion does not actually follow a Gaussian distribution, and higher order cumulants (Taylor series coefficients) are required to fully characterize the signal loss. In DTI, these higher order terms are ignored: the lower the b-value, the smaller these higher order terms become in relation to the diffusion tensor contribution to signal attenuation.

These results on the influence of the b-value also agree with the phantom experiments carried out by Laun et al. [48], where increases of the b-value were related to an underestimation of *λ*_1_ and an overestimation of *λ*_2_. However, this was only noticeable for b-values of 2000 *s*/*mm* ^2^ or higher, which is not the case in our experiments.

A second explanation for this issue, also related to the inaccuracies of the Gaussian model, can be found in the extracellular (free) water present in the voxels: the diffusion signal acquired is the combination of the signals coming from the water molecule inside myelinated axons (roughly Gaussian if they are coherently oriented) and from the water molecules outside these axons (free isotropic diffusion). The diffusivity of the free term is far higher, and accordingly the free water signal decays faster as the b-value increases. As a consequence, the larger the b-value the less relevant the contribution of the free water term. The measured MD will be a weighted average of the MDs of intra-cellular and extra-cellular water, hence for larger b-values the contribution of the intra-cellular water will gain more relevance pushing the overall MD towards its (smaller) value.

Although less relevant, there is also an effect of the different b-values on the average FA, mostly at high resolution. The effect of b-value over the FA has previously been studied by many authors [12, 29–31, 33], reporting non-significant differences. The relation between noise and FA caused by variations in b-values in simulations was examined in [32], and an underestimation of FA for *b* > 3000 *s*/*mm* ^2^ was found. However, there is no literature on the slight reduction of the average FA described in our results, which could be caused by eigenvalues repulsion or noise floor bias.

The visible differences between the effects of changes in the acquisition parameters on FA and MD are related to the fact that they measure different things. FA measures the ratio of magnitudes of the anisotropic component of the the diffusion tensor; while MD measures the total amount of diffusivity within a voxel and doesn’t have a directional component.

Because of this, an increase in the angular resolution due a higher number of gradients will affect the FA result. At the same time, a stronger b-value will specially affect the MD results, because it causes an increase of the overall displacement of the water molecules in all directions. The effects of spacial resolution on both measures are not as clear and should be focused in more detail in future studies.

Finally, we want to recall that two different **tractography** approaches have been employed in the experiments carried out in this paper, and some differences between the two methods appear in the results. Consider, for instance, results in Fig 4(a) and 4(b), showing a higher average FA when using the RKT tractography method than for GT. This fact is related to the differences between the behavior of both algorithms in terms of number of fibers, fiber length and total volume.

An important part of the differences between the RKT and GT reconstructions can be attributed to the spread of fibers in the later. RKT tends to concentrate tracks in high anisotropy zones, being able to follow sharper angles; GT tracks, on the other hand, creates smoother tracts and does not follow as closely the underlying DTI infomation.

Indeed, the RKT algorithm produces, at least for a high number of gradient directions, many more fibers than the GT method (see Fig 2(c) and 2(d)). These fibers are, however, slightly shorter on average (Fig 2(a) and 2(b)), possibly indicating that the resulting fiber bundle covers less white matter area. This is confirmed by Fig 3, where the RKT method shows a smaller total volume in the fiber bundle. Together, these figures indicate that fibers obtained using GT reach further than RKT in white matter areas with lower FA, which in turn explains the lower average FA for this particular tractography method.

GT reconstructions tend to be longer and less variable. The number of reconstructed tracks that can complete the path between two target ROIs in complex zones is higher and less affected by acquisition parameters. These differences can be specially appreciated in high resolution reconstructions.

While there are indeed considerable differences between both tractography methods, the key point is that their behavior with respect to changes in the acquisition parameters follow the same pattern. Many different tractography algorithms can be used for DTI analysis, and therefore it is not possible to guarantee that every tractography method will exactly follow the patterns described here. However, understanding the effect of changes in the acquisition parameters on the resulting DTI volume and the related scalar parameters will help understand as well the behavior of the tractography methods that operate on this DTI information.

## Conclusion

Changes in the acquisition parameters in DTI are known to have an important effect on the values of the scalar parameters that are commonly studied in white matter analysis, as has been shown in a growing number of studies in the literature. Considering that tractography-based approaches represent a substantial share of these type of clinical studies, this paper is devoted to the analysis of the influence of acquisition parameters in a tractography-based setting, with a focus on the consequences of changes in multiple parameters: number of gradient directions, b-value and voxel resolution.

Results from our experiments pointed out that, while the number of gradient directions and the voxel resolution greatly affect the FA, AD and RD indexes, variations on the b-value specially affect the MD, but also AD and RD. Therefore, no single scalar measure is robust to changes in all the considered acquisition parameters, although each of them is relatively insensitive to changes in some of the parameters.

Relevant differences were also found between the behavior of the two tractography algorithms employed, in terms of number of fibers, average fiber length, tractography volume and volume overlap. These differences, in turn, cause differences in the final scalar measures but, in spite of these differences, changes in the acquisition parameters affect scalar measures in the same way, regardless of the tractography method employed.

## Supporting Information

S1 File Main Findings. Summary of the main findings described in this paper.
(PDF)

S2 File MATLAB supplementary data. MATLAB data of the experiments described in this paper.
(ZIP)

S3 File More tables and figures. Additional results not shown in this paper.
(PDF)
