# Supplementary material for: Impact of MR Acquisition Parameters on DTI Scalar Indexes: A Tractography Based Approach
Source: PLoS One. 2015 Oct 12;10(10):e0137905. doi: 10.1371/journal.pone.0137905 (PMC4601730; doi:10.1371/journal.pone.0137905)
Supplement: S1 File — Summary of the main findings described in this paper. (PDF) [file pone.0137905.s001.pdf]

Supplementary material

Main findings presented on this paper,
compared with previous literature.

	$\uparrow \Delta B$ (increasing from $b = 800$ to $b = 1300\ s/m^2$)
FA	Small but not-significant decrease in mean values of FA, specially on High-Resolution reconstructions. These is similar to findings in [1],[7].
MD	Significant linear decrease in mean values of MD. This effect was expected, as reported in [5],[8],[4], in both simulations and real data.
AD	Significant decrease in mean values of AD. This effect was reported in [2] **(in rats)** and [8] **(in simulations)**.
RD	No significant difference in the mean of RD values. Decrease of the std. of RD values in the GT rec. **Our results differ from** those of [2], who reported a decrease of the mean RD (in rats).

	$\uparrow \Delta R$ (increasing from $r = 2$ to $r = 3\ mm^3$)
FA	Significant increase of mean FA. **Previously unreported effect for different isotropic voxel resolutions**.
MD	Small but not-significant increase on mean MD values. Significant decrease in the std. of MD. [8] reported a decrease of the mean values of MD in simulations. [9] reported no differences on mean MD values between different resolutions.
AD	Significant increase in mean values of AD, specially for a very low number of gradients reconstructions. **This is a previously unreported effect.**
RD	Significant increase in mean values of RD, specially for a very low number of gradients reconstructions. Significant increase on the std. values of RD. **This is a previously unreported effect.**

	$\uparrow \Delta G$ (increasing from $G = 6$ to $G = 61$ dirs.)
FA	Remarkable ang significant decrease on the mean values of FA This effect has been reported by [3],[6],[10][11]
MD	Significant differences between the $G = 6$ and the rest of reconstructions. **This is a previously unreported effect.**
AD	Significant increase on the mean values of AD. **This a previously unreported effect.**
RD	Significant decrease on the mean values of RD. **This a previously unreported effect.**

* p-value threshold for significance $0'01$

[1] Bisdas, S., Bohning, D., Besenski, N., Nicholas, J., Rumboldt, Z., 2008. Reproducibility, interrater agreement, and age-related changes of fractional anisotropy measures at 3T in healthy subjects: effect of the applied b-value. Am. J. Neuroradiol 29, 1128–1133.

[2] Hui, E., Cheung, M., Chan, K., Wu, E., 2010. B-value dependence of DTI quantitation and sensitivity in detecting neural tissue changes. Neuroimage 49, 2366–2374.

[3] Jones, D., 2004. The effect of gradient sampling schemes on measures derived from diffusion tensor MRI: a Monte Carlo study. Neuroimage 51, 807–815.

[4] Jones, D., Basser, P., 2004. Squashing peanuts and smashing pumpkins: how noise distorts diffusion-weighted MR data. Magn. Reson. Med 52, 979–993.

[5] Jones, D., Cercignani, M., 2010. Twenty-five pitfalls in the analysis of diffusion mri data. NMR Biomed. 23, 803–820.

[6] Landman, B., Farrell, J., Jones, C., 2007. Effects of diffusion weighting schemes on the reproducibility of DTI-derived fractional anisotropy, mean diffusivity, and principal eigenvector measurements at 1.5T. Neuroimage 36, 1123–1138.

[7] Melhem, E., Itoh, R., Jones, L., Barker, P., 2000. Diffusion tensor MR imaging of the brain: effect of diffusion weighting on trace and anisotropy measurements. Am. J. Neuroradiol 21, 1813–1820.

[8] Metzler-Baddeley, C., O'Sullivan, M., Bells, S., Pasternak, O., Jones, D., 2012. How and how not to correct for CSF-contamination in diffusion MRI. Neuroimage 59, 1394–1403.

[9] Takao, H., Hayashi, N., Inano, S., Ohtomo, K., 2011. Effect of head size on diffusion tensor imaging. Neuroimage 57, 958–967.

[10] Wang, J., Abdi, H., Bakhadirov, K., Diaz-Arrastia, R., Devous, M., 2012. A comprehensive reliability assessment of quantitative diffusion tensor tractography. Neuroimage 60, 1127–1138.

[11] Zhan, L., Chiang, M.C., Barysheva, M., Toga, A., McMahon, K., 2008. How many gradients are sufficient in high-angular resolution diffusion imaging (HARDI)?, in: Workshop on Computational Diffusion MRI, MICCAI 2008. New York, USA., pp. 216–224.
