# Supplementary material for: Impact of MR Acquisition Parameters on DTI Scalar Indexes: A Tractography Based Approach
Source: PLoS One. 2015 Oct 12;10(10):e0137905. doi: 10.1371/journal.pone.0137905 (PMC4601730; doi:10.1371/journal.pone.0137905)
Supplement: S3 File — Additional results not shown in this paper. (PDF) [file pone.0137905.s003.pdf]

Additional material for paper

Impact of MR acquisition parameters on DTI scalar indexes: a tractography based approach

G. Barrio-Arranz, R. de Luis-García, A. Tristán-Vega,
M. Martín-Fernández, S. Aja-Fernández

LPI, Universidad de Valladolid, Spain

Figures and results for all the bundles considered

1- List of Bundles considered in the experiment:

- Corpus Callosum genu (CCG)
- Corpus Callosum anterior (CCA)
- Corpus Callosum posterior (CCP)
- Corpus Callosum splenium (CCS)
- Left cingulum (CGL)
- Right cingulum (CGR)

2- Scalar indices

- FA: Fractional Anisotropy
- MD: Mean Diffusivity
- AD: Axial Diffusivity (first eigenvalue)
- RD: Radial diffusivity (mean of 2nd and 3rd eigenvalues)
- AM, MO: Anisotropy Mode*

*Ennis DB, Kindlmann G. 'Orthogonal tensor invariants and the analysis of diffusion tensor magnetic resonance images'. Magnetic Resonance in Medicine;55 (1):136-146 (2006).

1 – Genu of the Corpus Callosum (CCG)

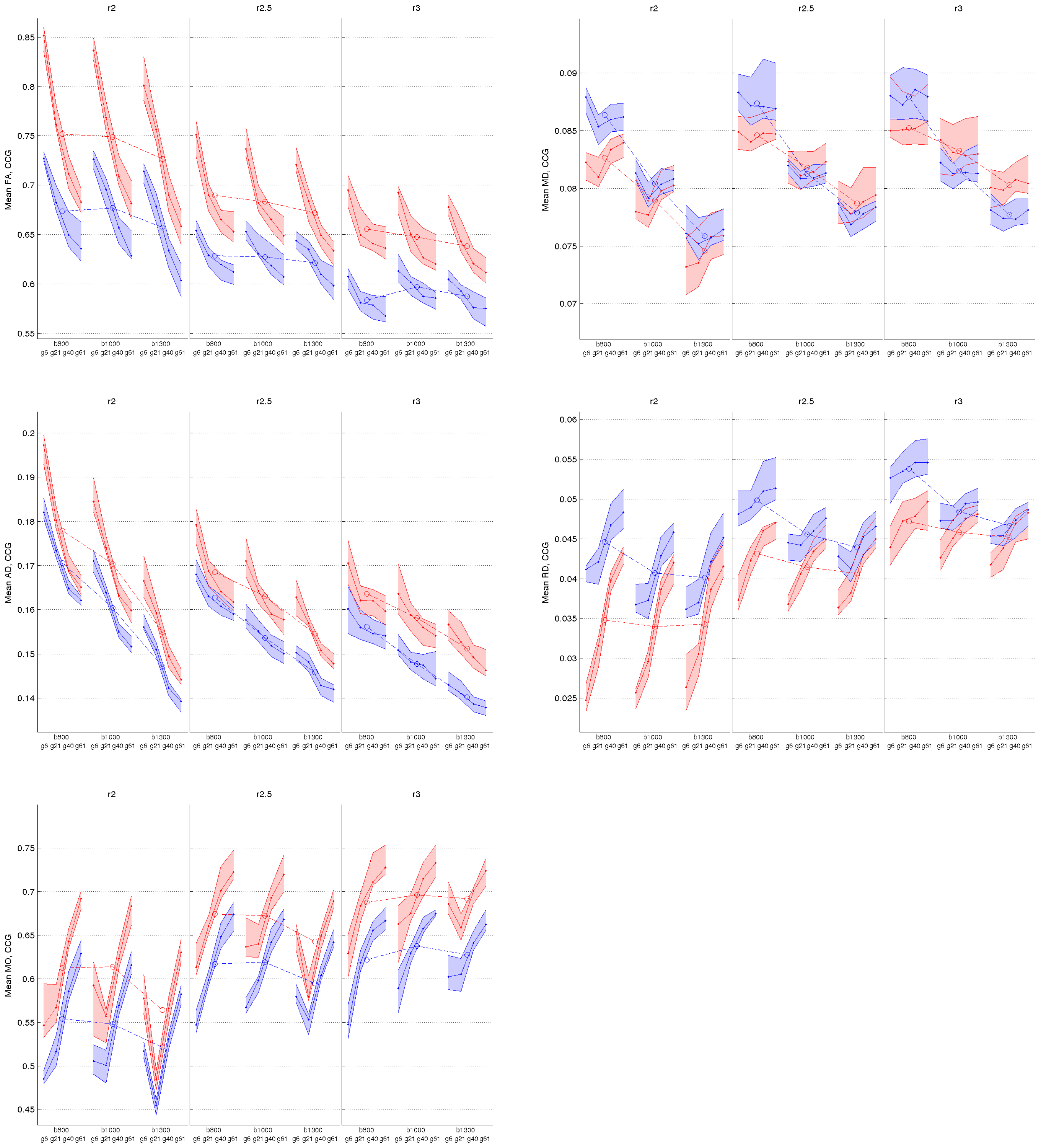

Figure 1: Average values for different scalar indices on the CCG.

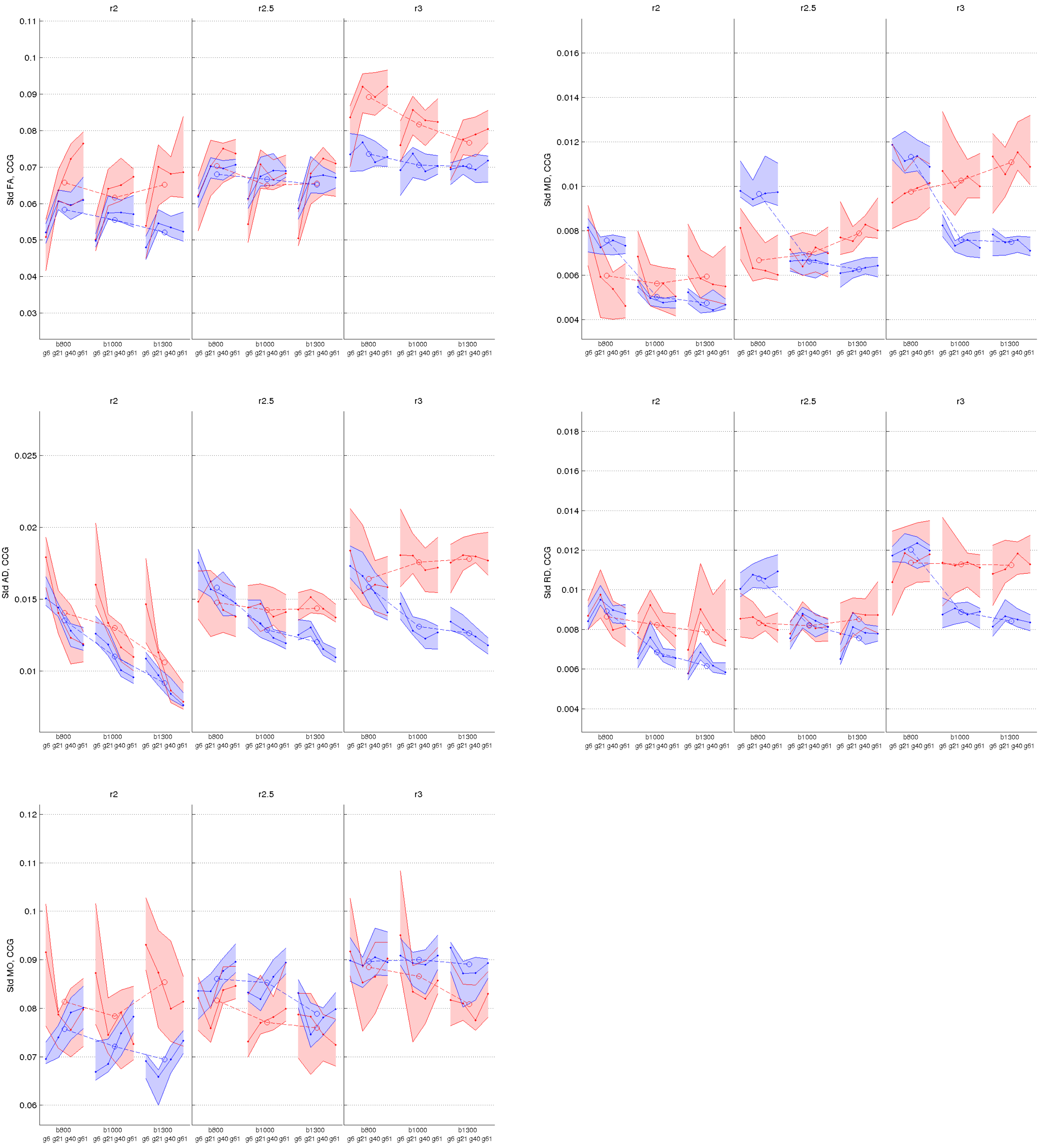

Figure 2: Standard dev. values for different scalar indices on the CCG.

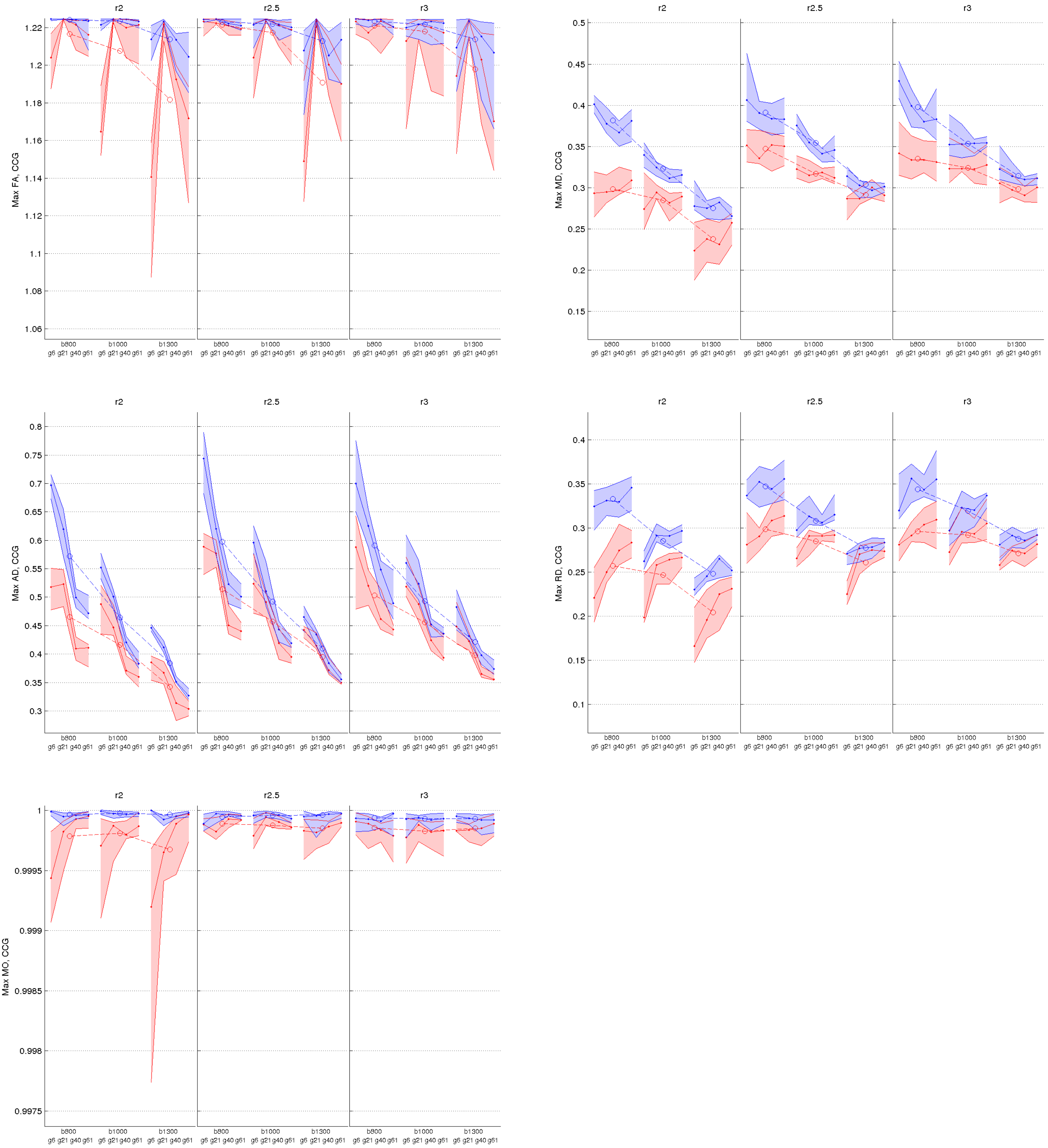

Figure 3: Maximum values for different scalar indices on the CCG.

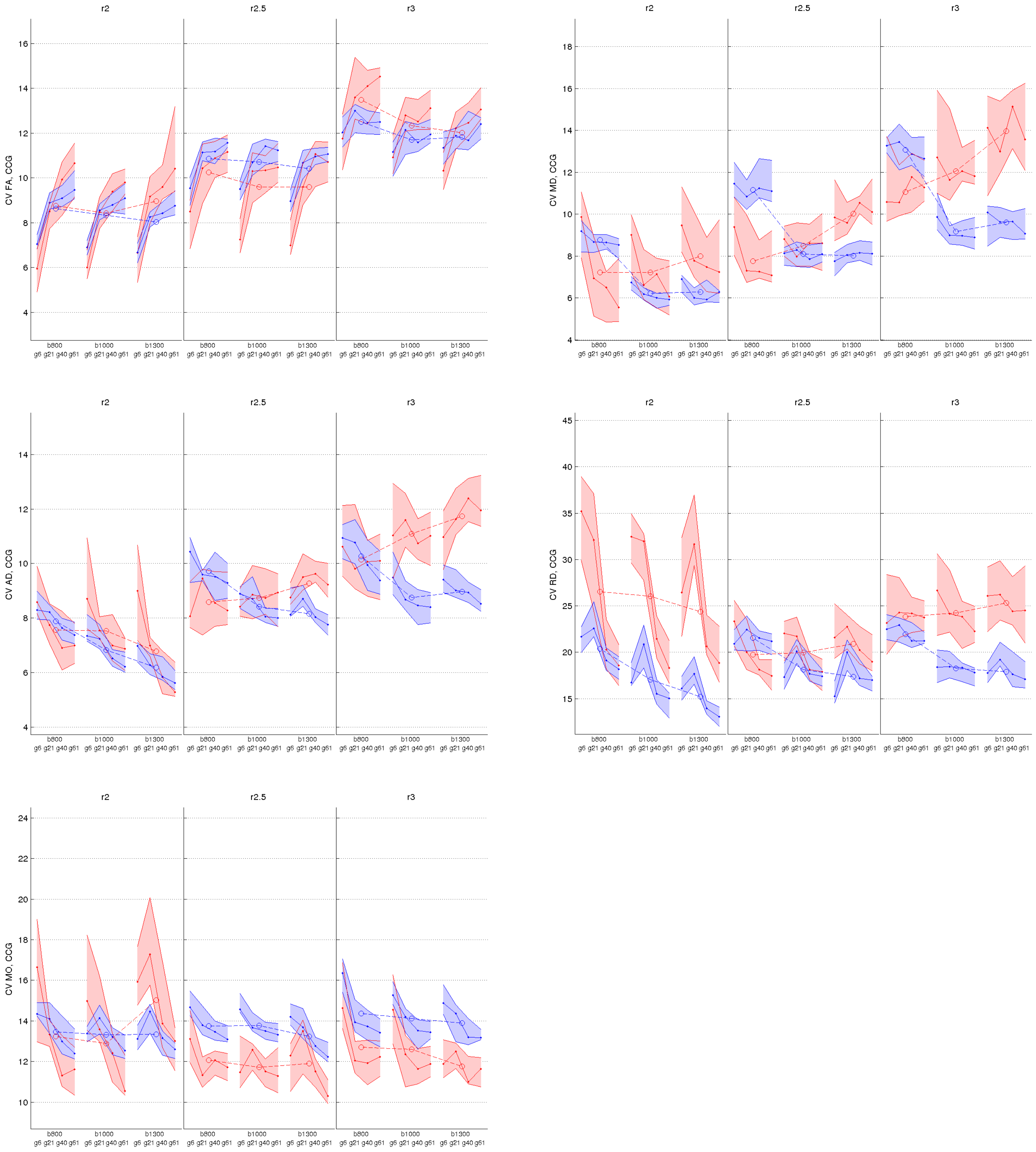

Figure 4: Coefficient of Variation values for different scalar indices on the CCG.

2 – Corpus Callosum anterior (CCA)

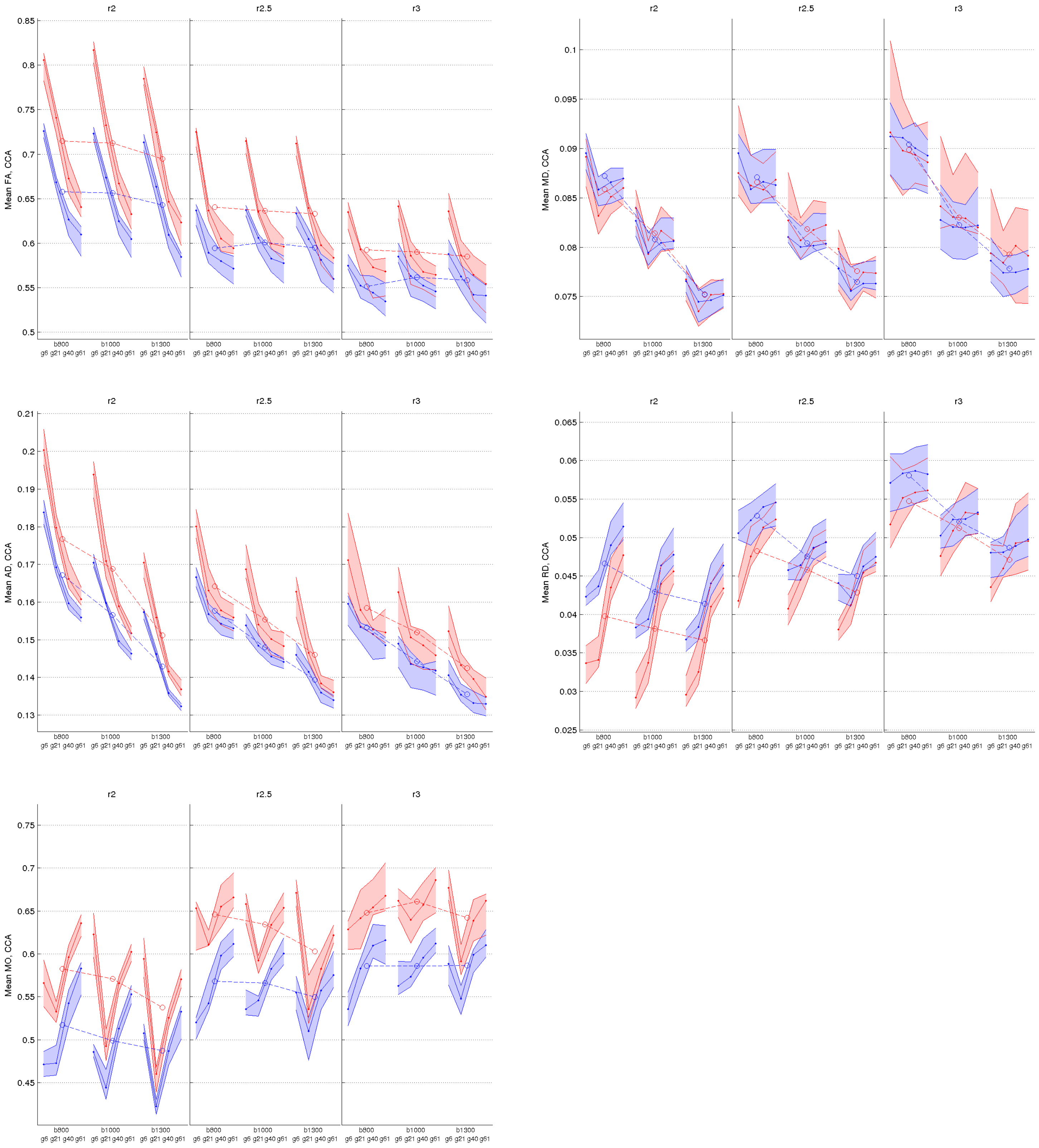

Figure 5: Average values for different scalar indices on the CCA.

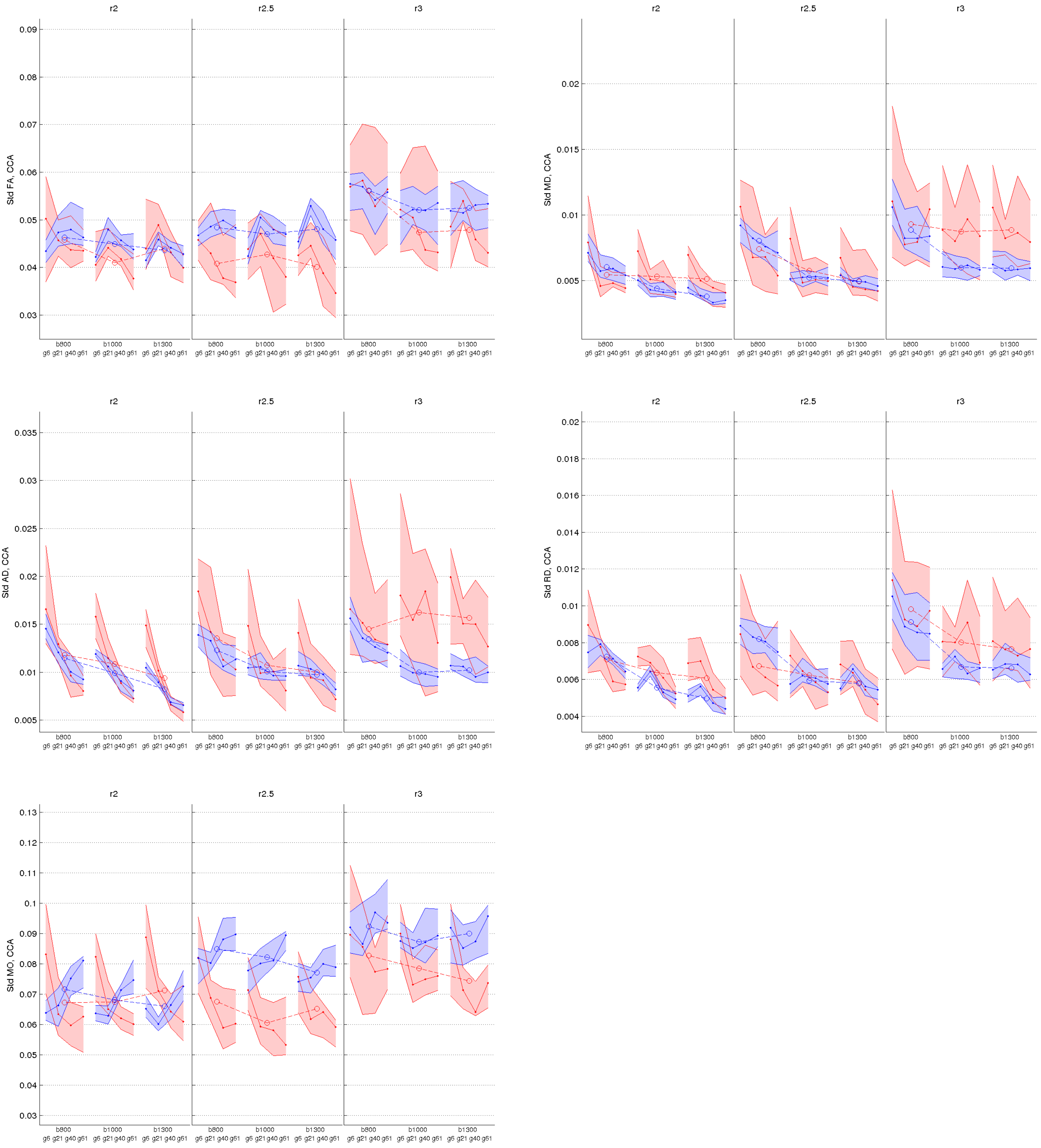

Figure 6: Standard dev. values for different scalar indices on the CCA.

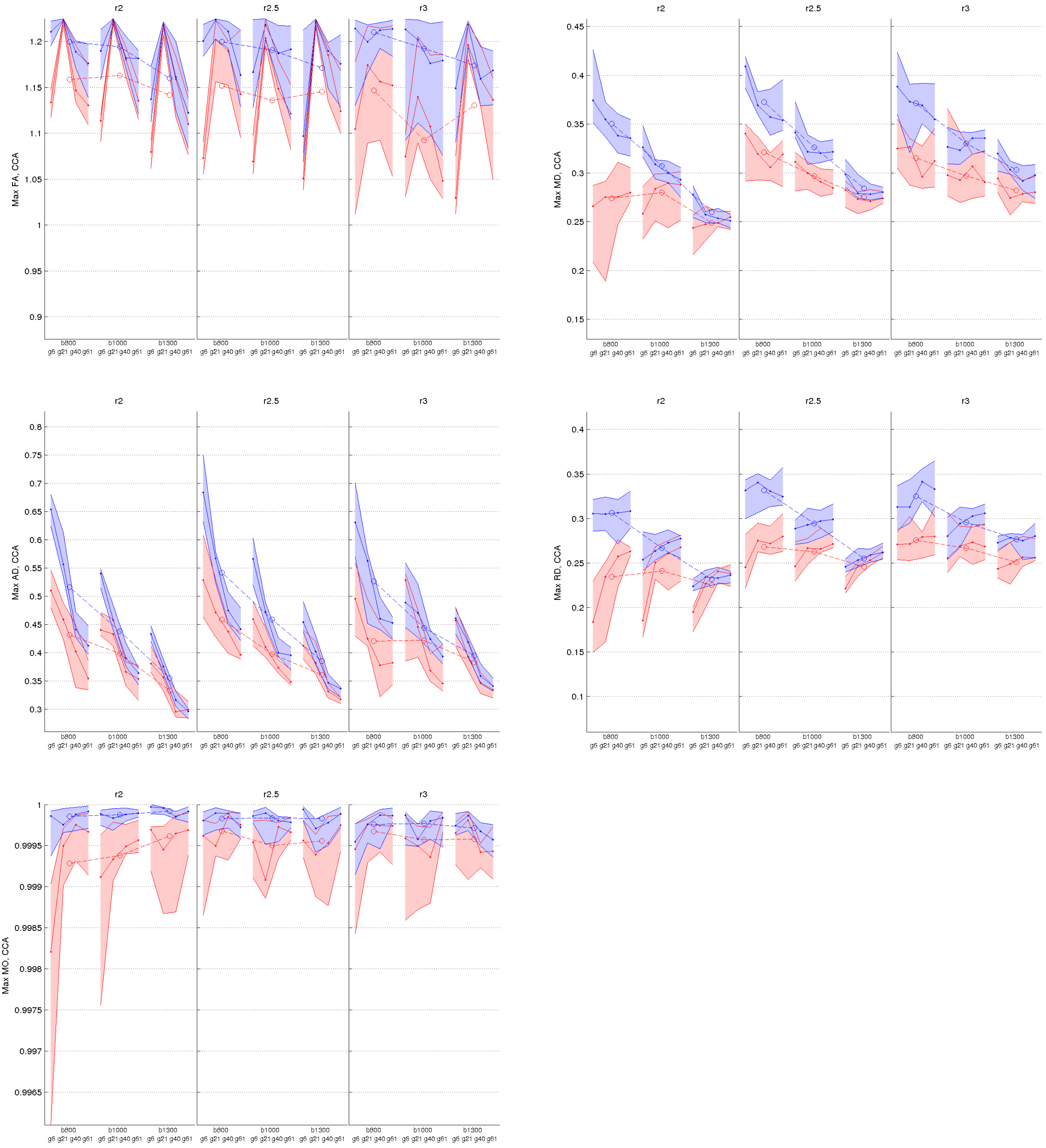

Figure 7: Maximum values for different scalar indices on the CCA.

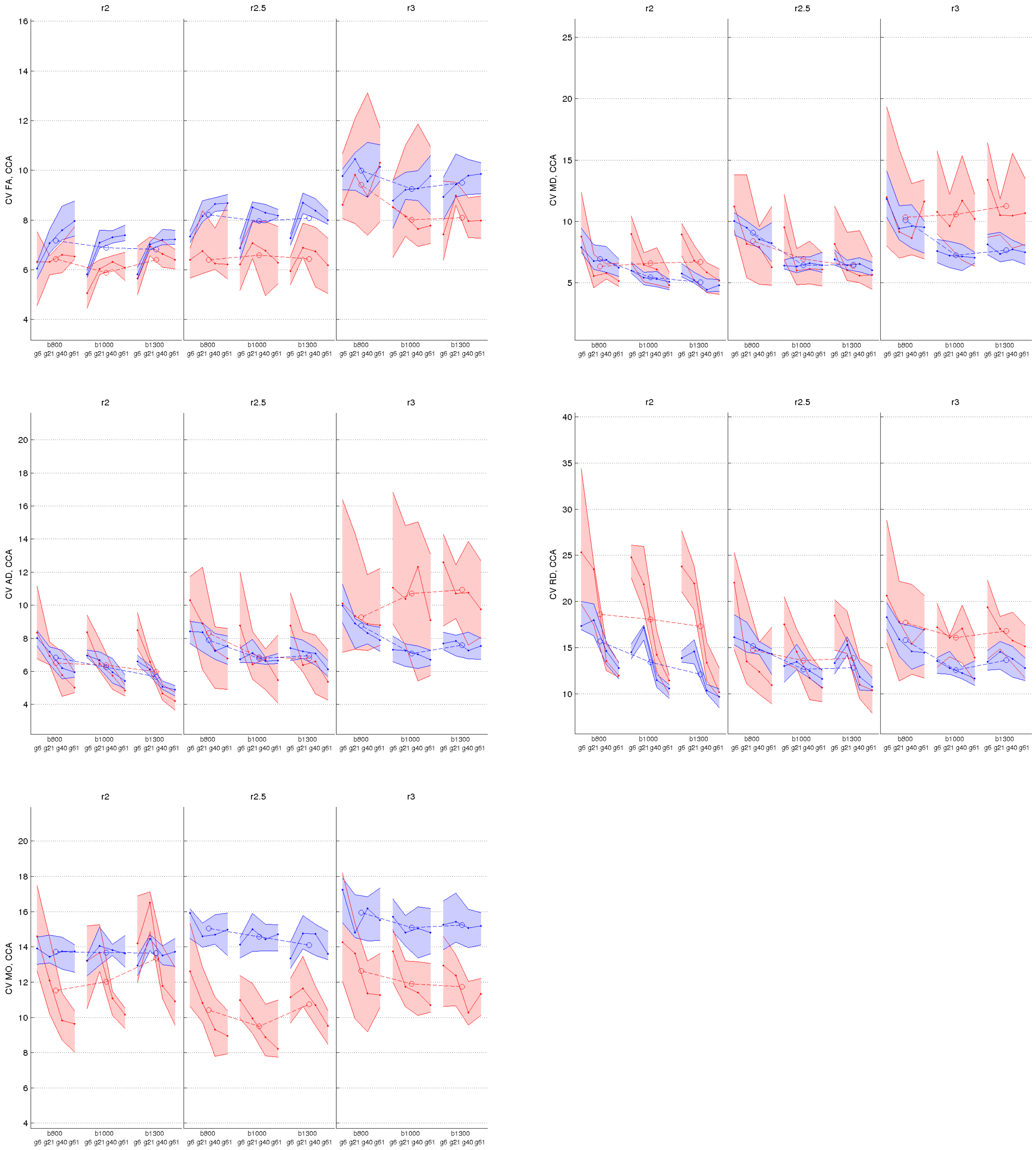

Figure 8: Coefficient of Variation values for different scalar indices on the CCA.

3 – Corpus Callosum posterior (CCP)

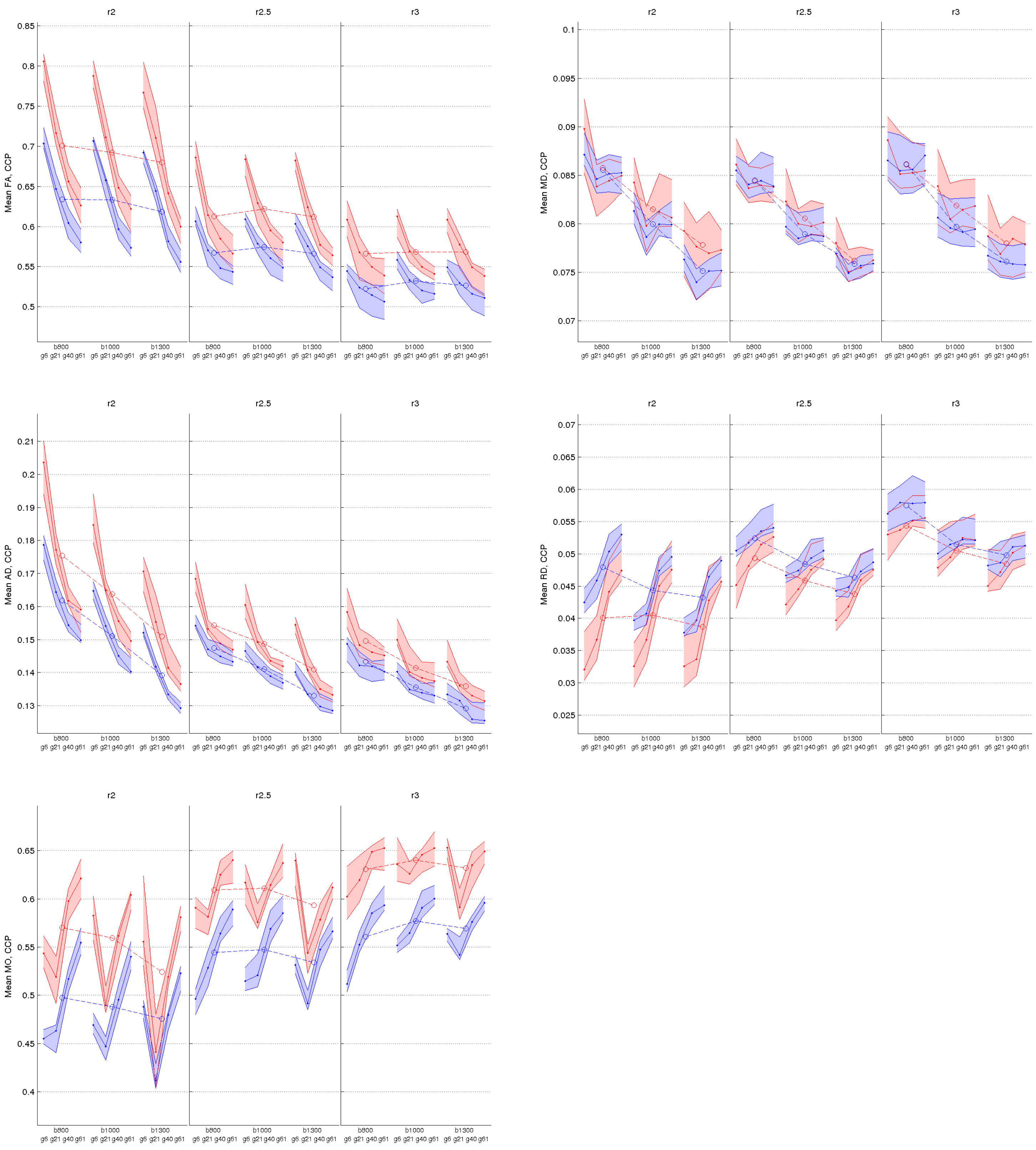

Figure 9: Average values for different scalar indices on the CCP.

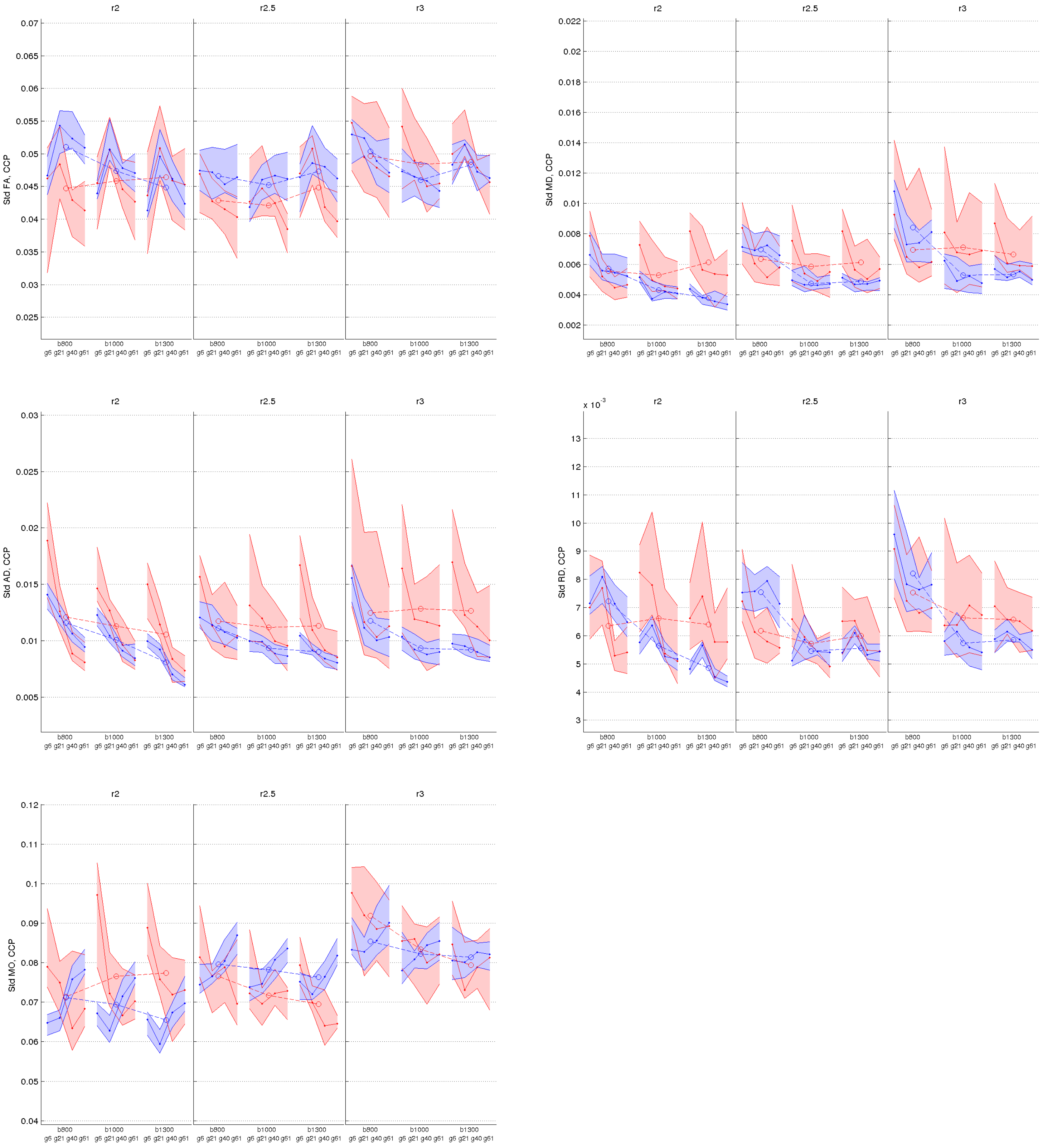

Figure 10: Standard dev. values for different scalar indices on the CCP.

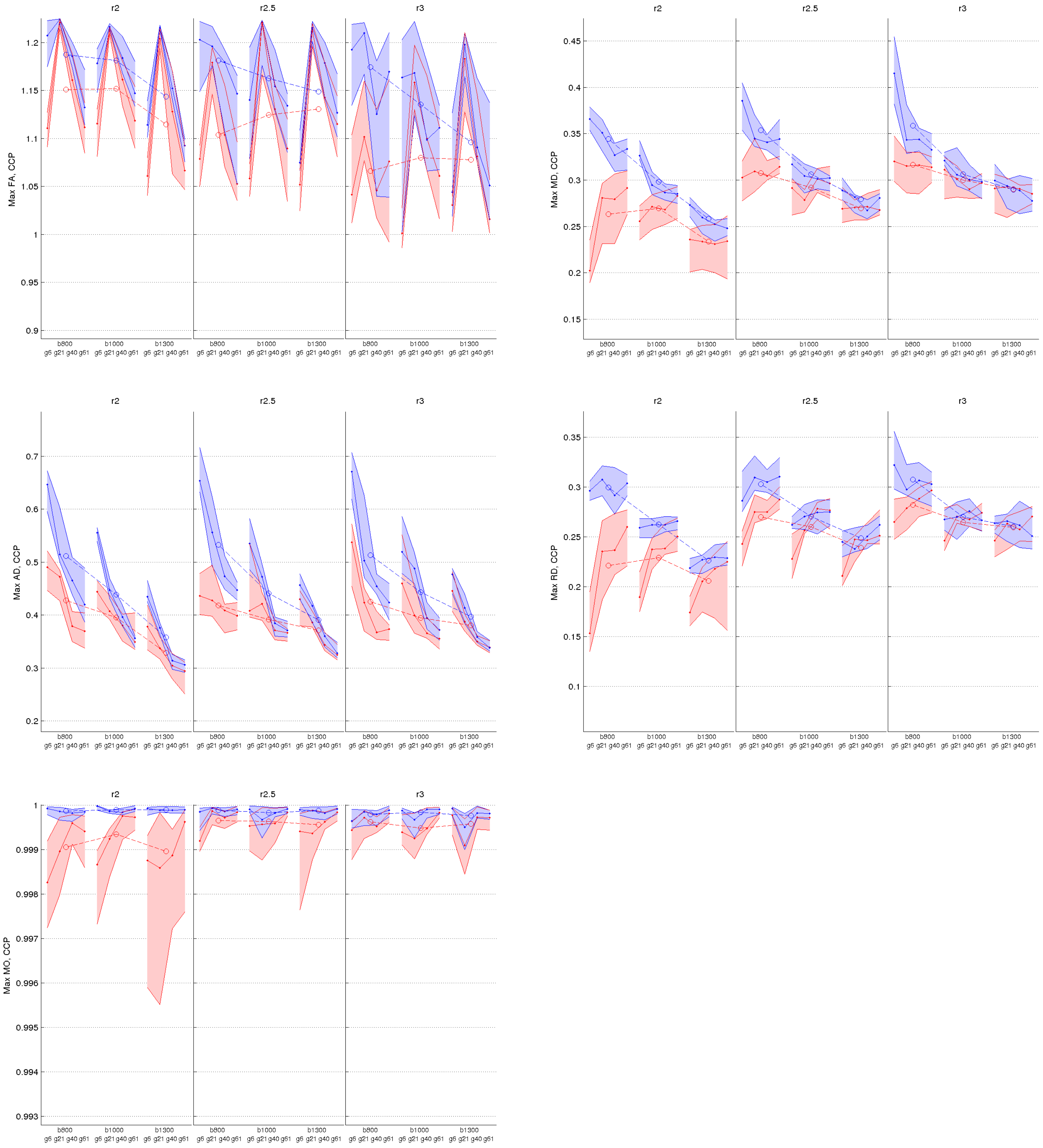

Figure 11: Maximum values for different scalar indices on the CCP.

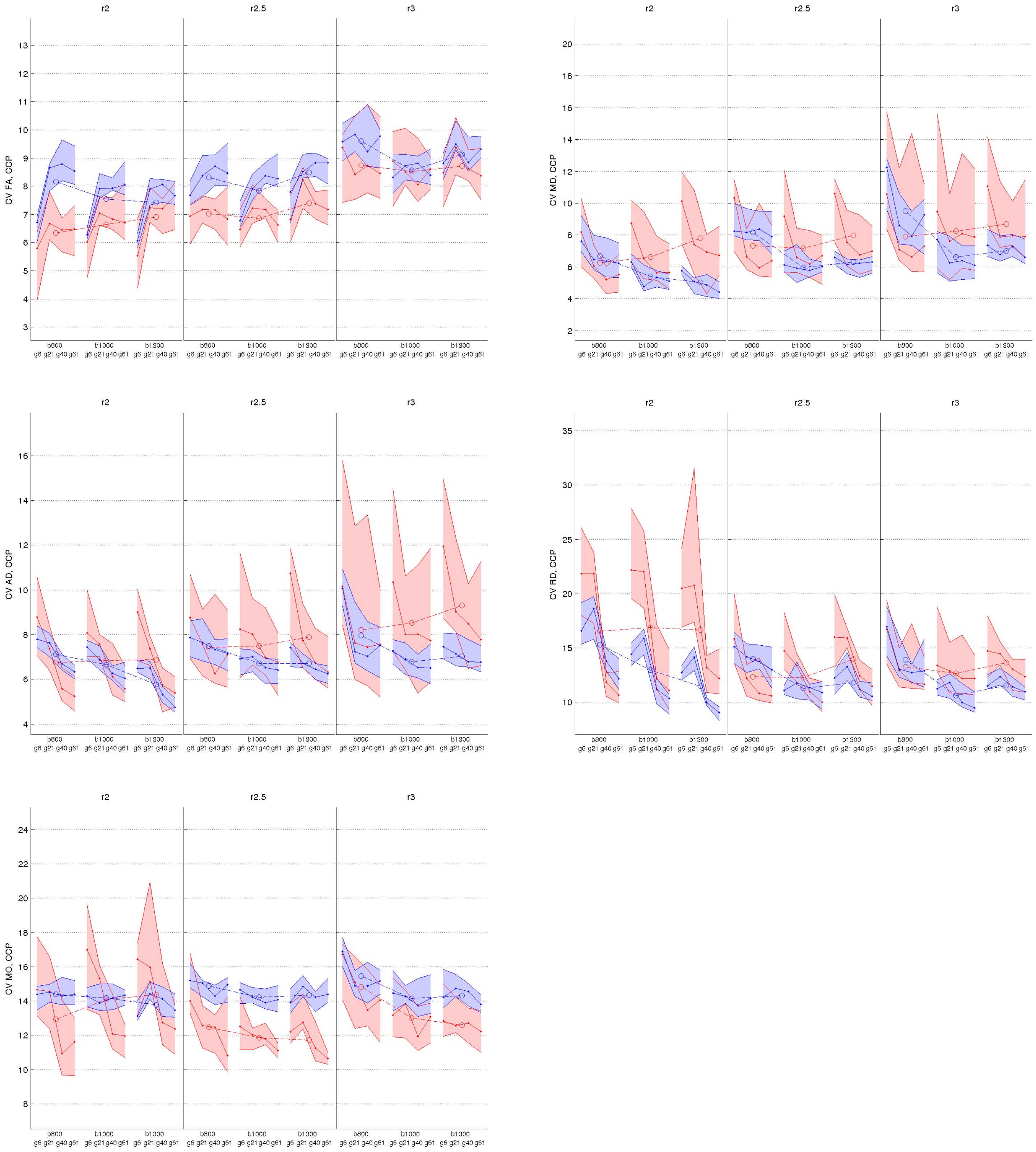

Figure 12: Coefficient of Variation values for different scalar indices on the CCP.

4 – Splenium of the Corpus Callosum (CCS)

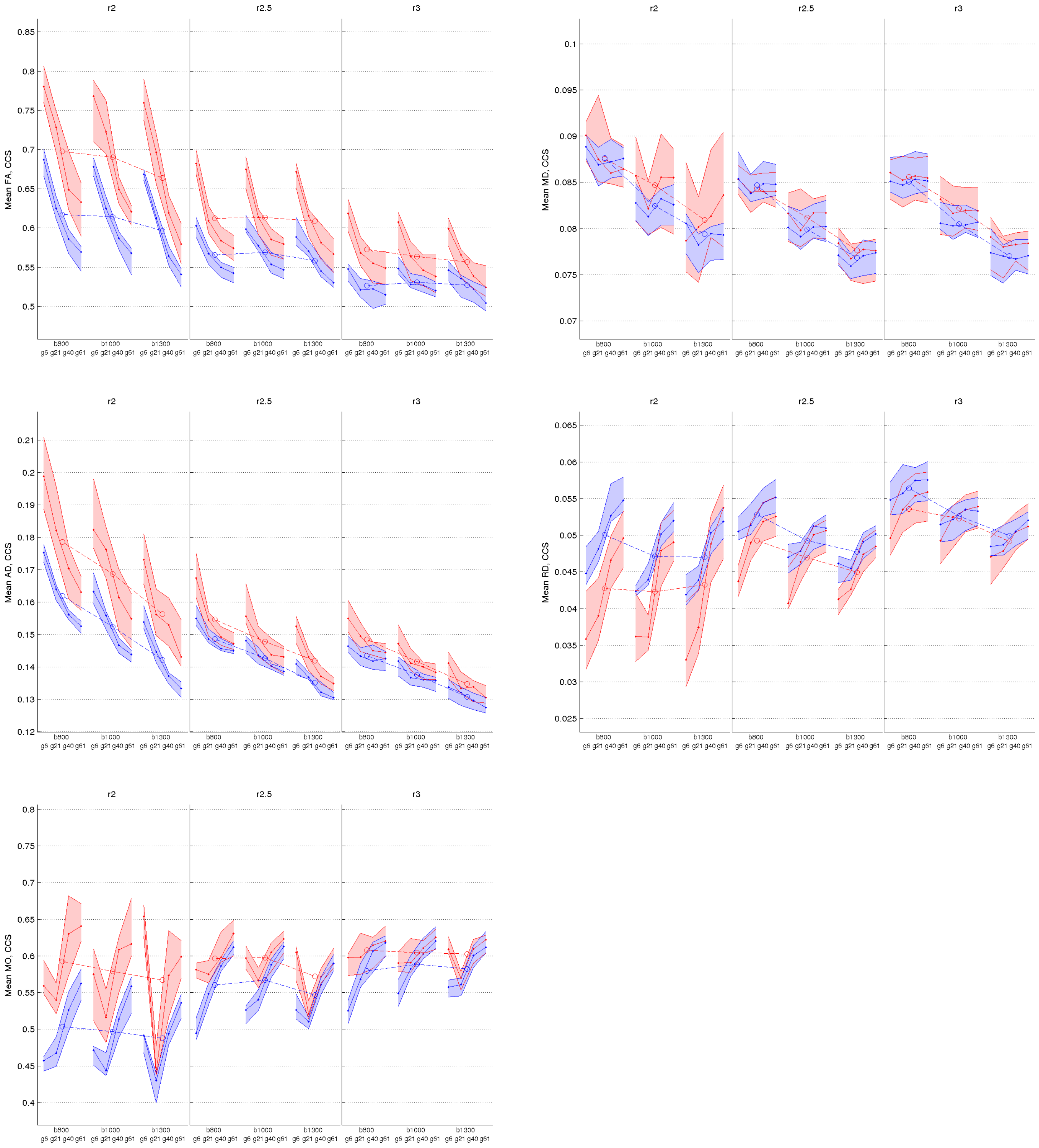

Figure 13: Average values for different scalar indices on the CCS.

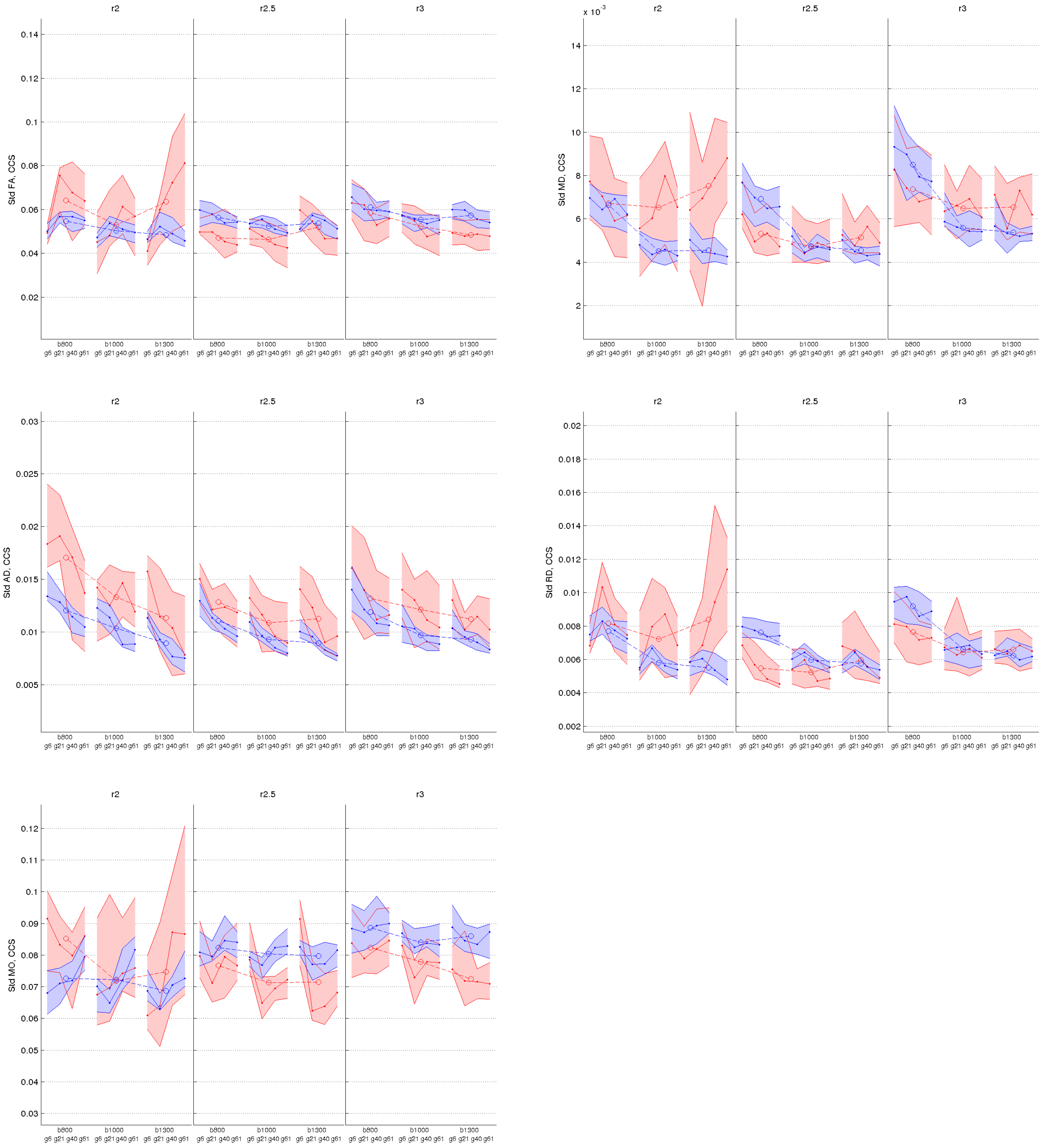

Figure 14: Standard dev. values for different scalar indices on the CCS.

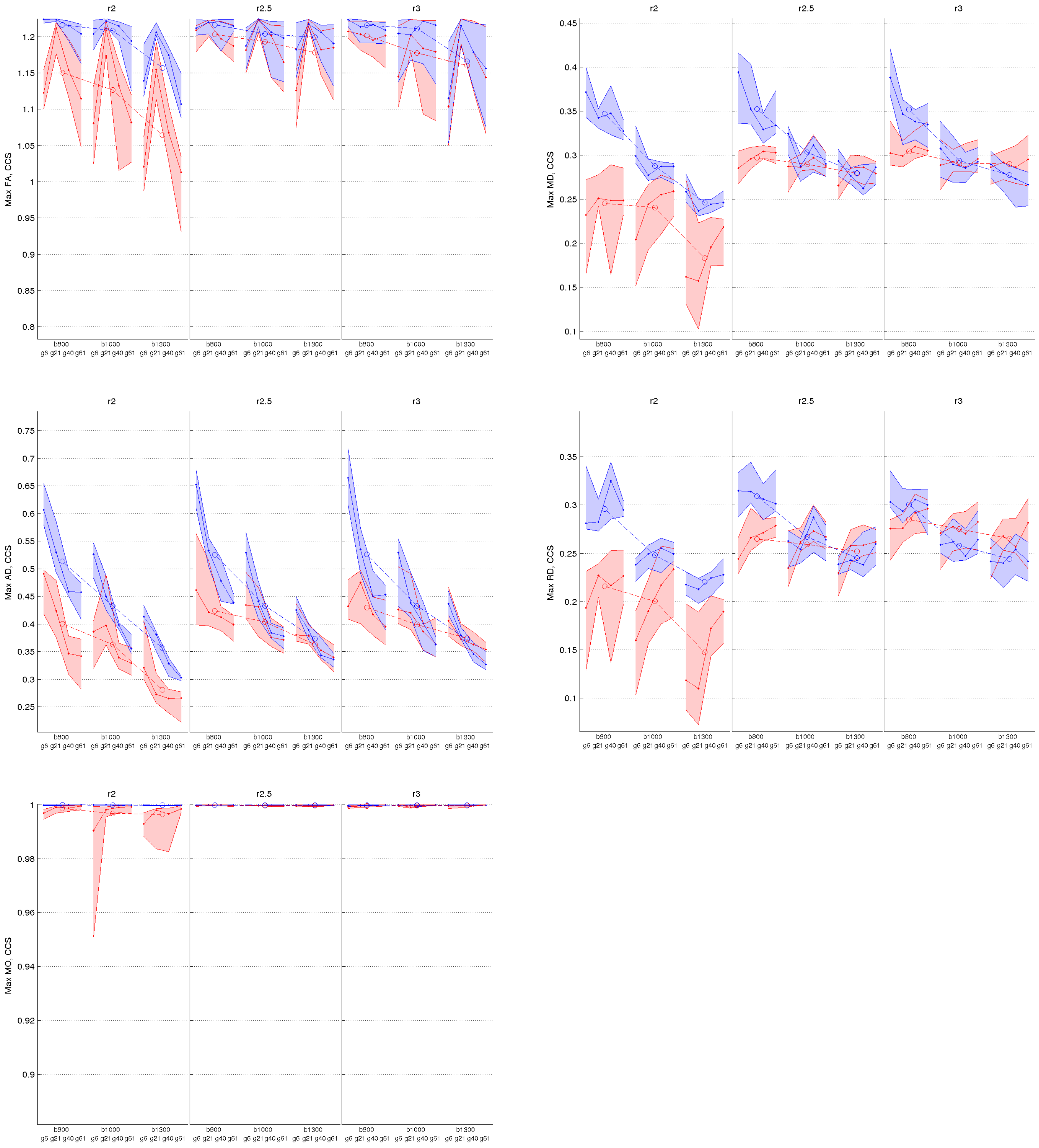

Figure 15: Maximum values for different scalar indices on the CCS.

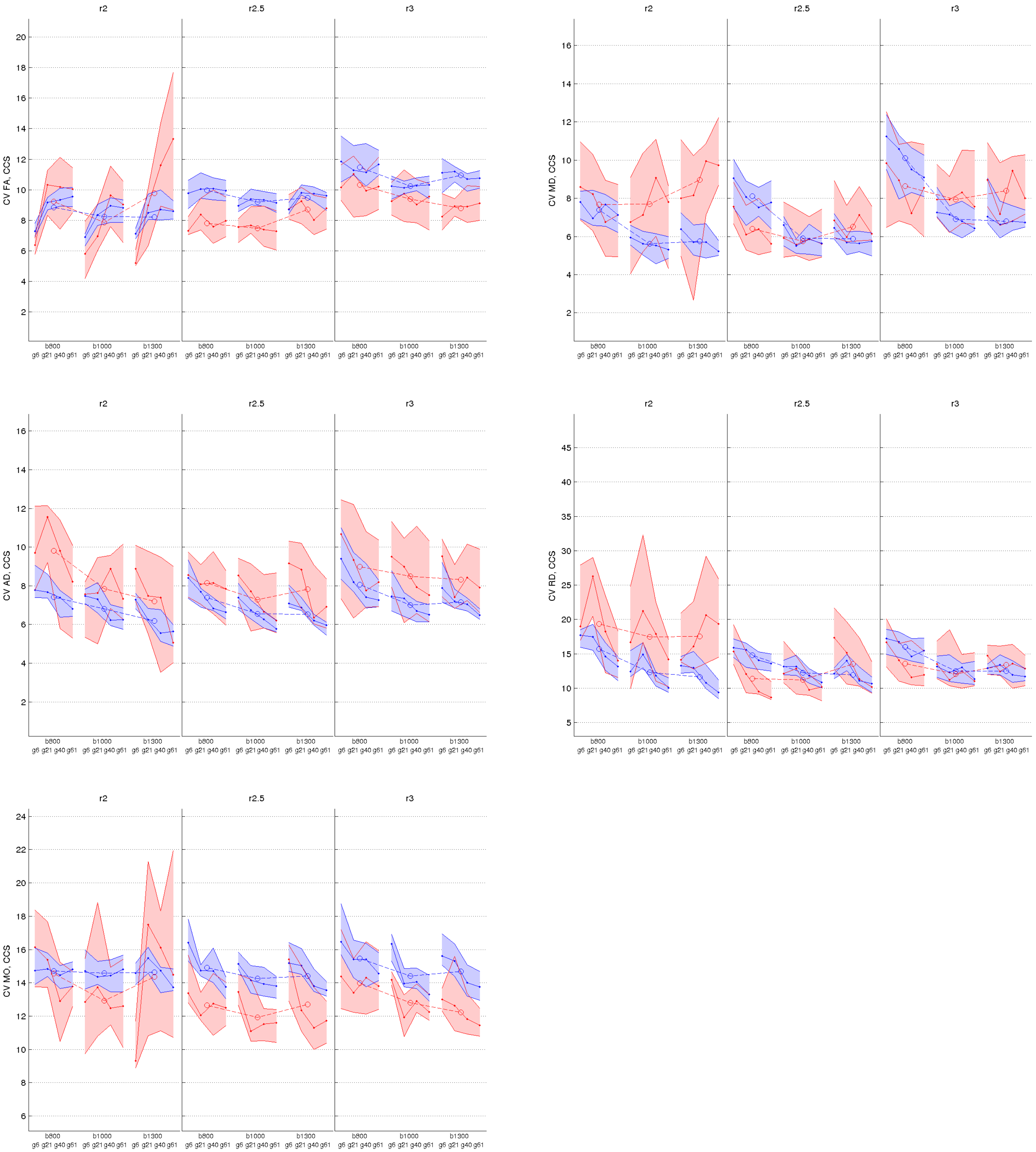

Figure 16: Coefficient of Variation values for different scalar indices on the CCS.

5 – Left Cingulum (CGL)

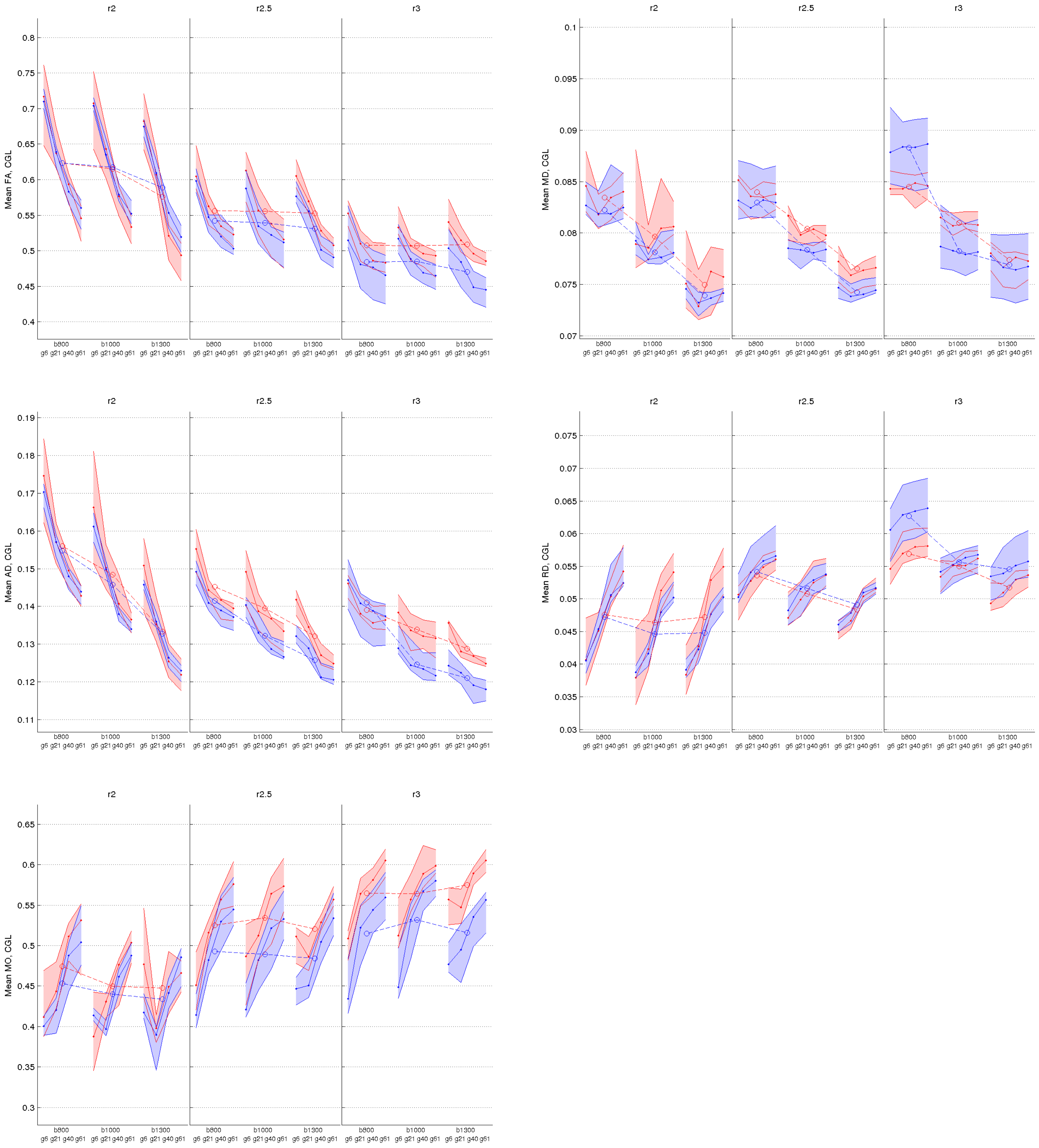

Figure 17: Average values for different scalar indices on the CGL.

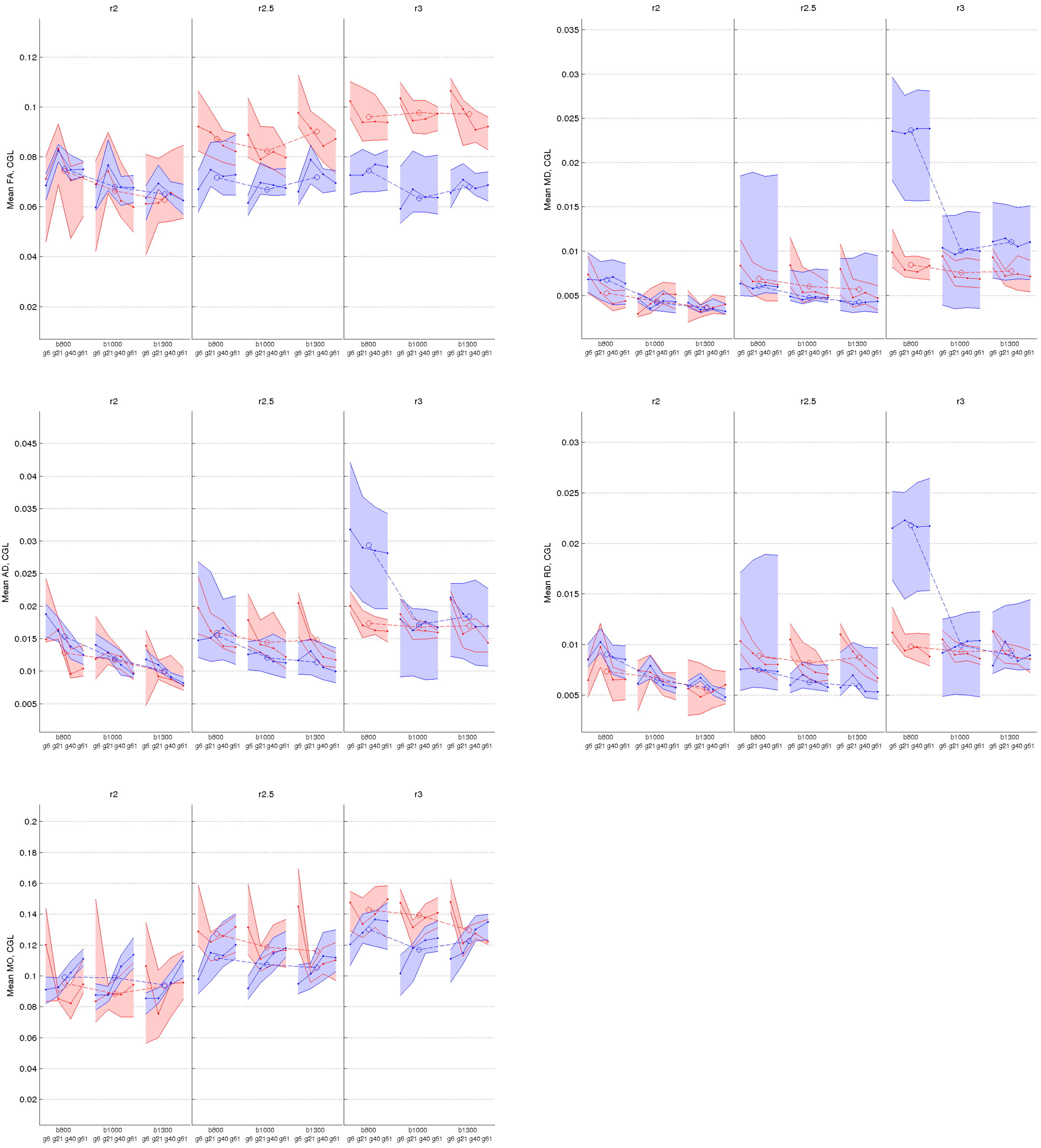

Figure 18: Standard dev. values for different scalar indices on the CGL.

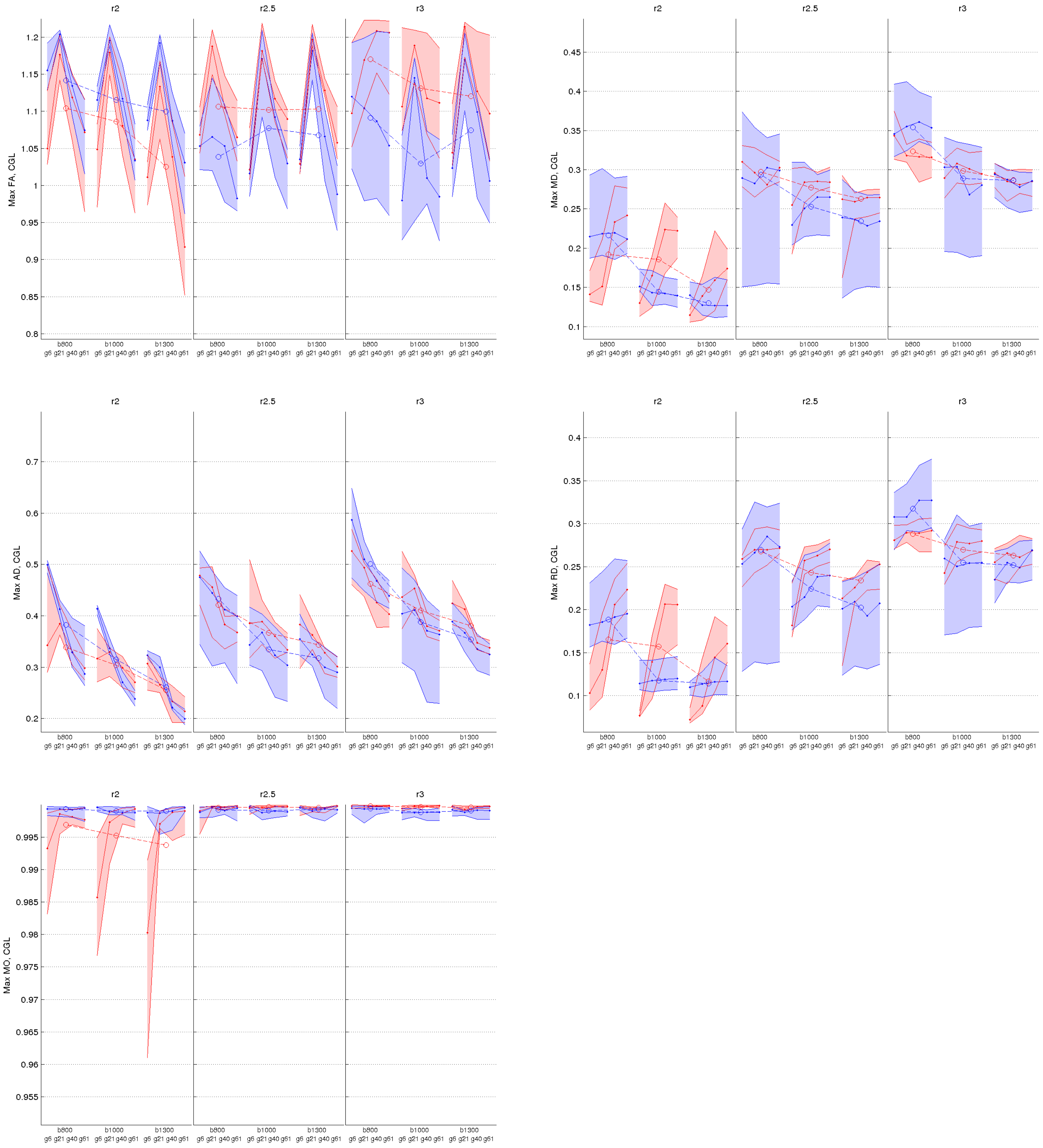

Figure 19: Maximum values for different scalar indices on the CGL.

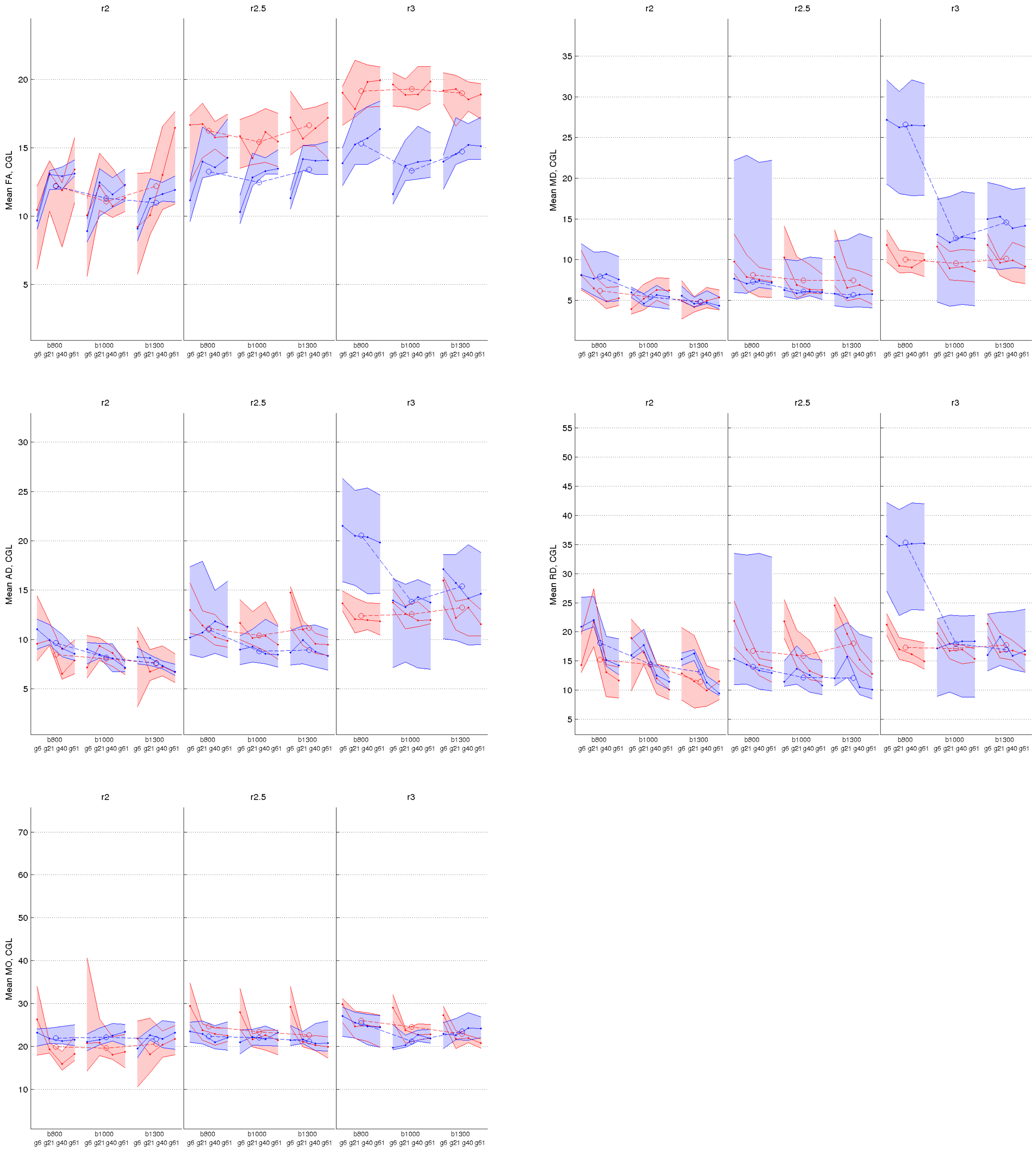

Figure 20: Coefficient of Variation values for different scalar indices on the CGL.

6 – Right Cingulum (CGR)

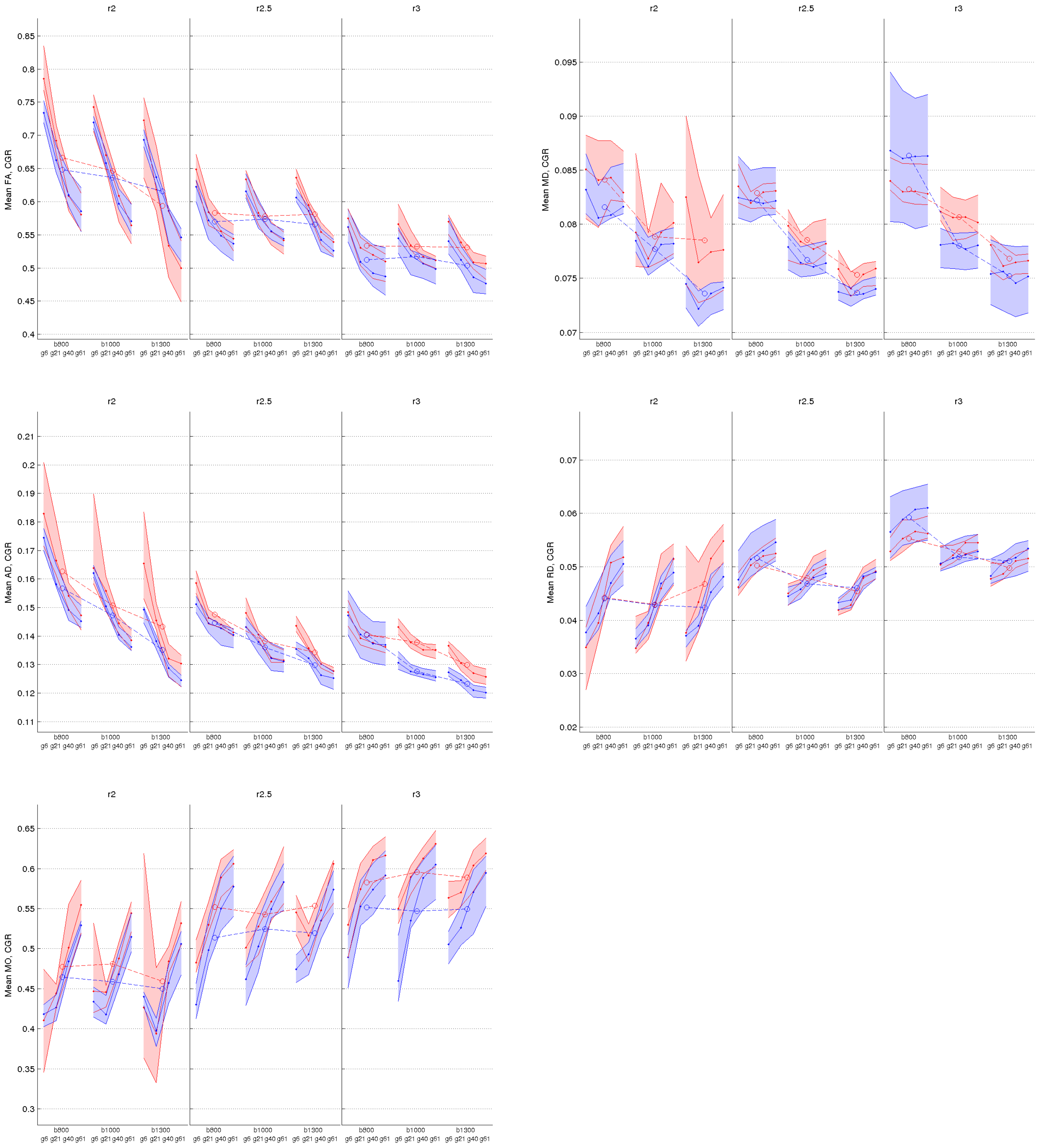

Figure 21: Average values for different scalar indices on the CGR.

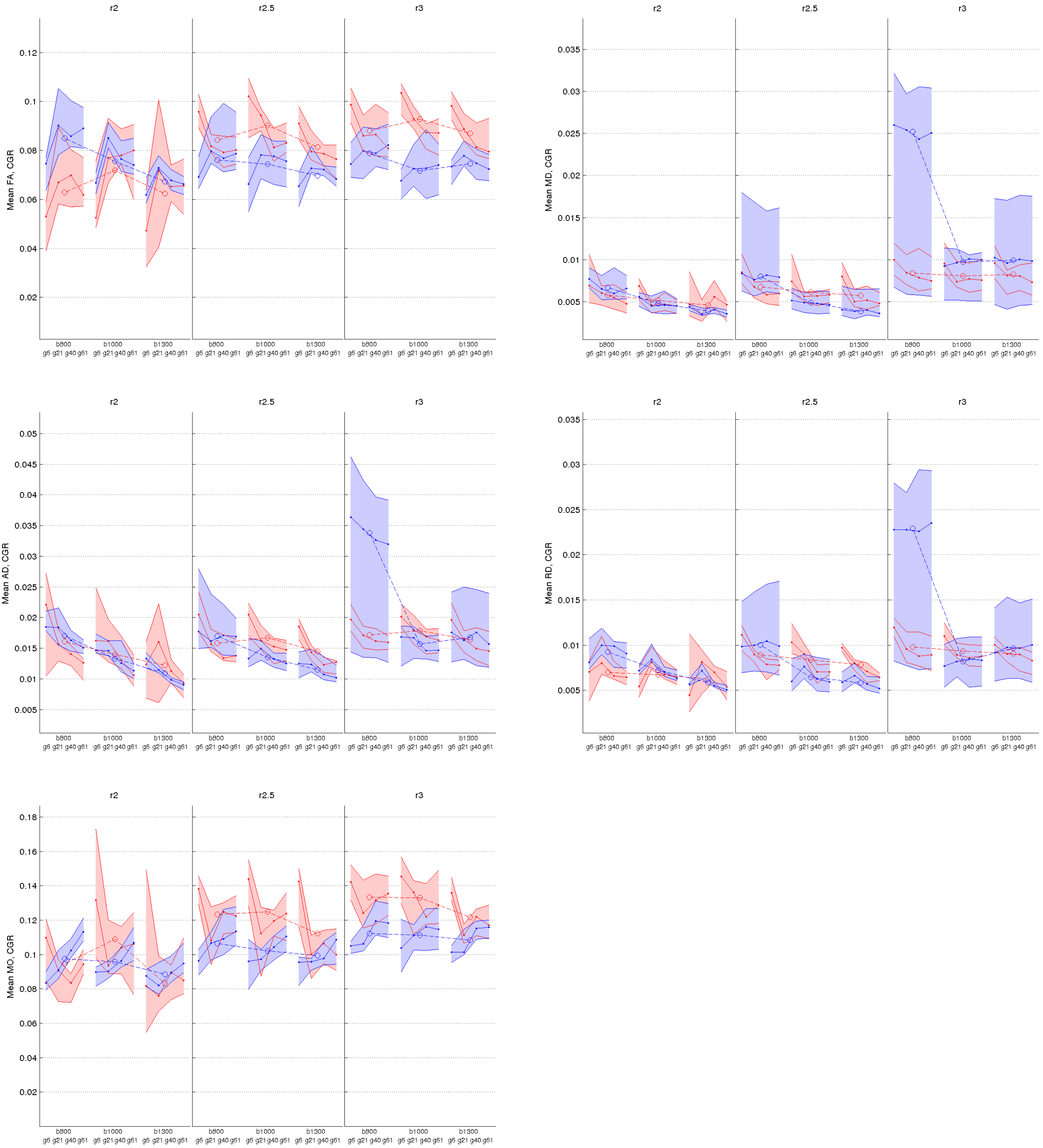

Figure 22: Standard dev. values for different scalar indices on the CGR.

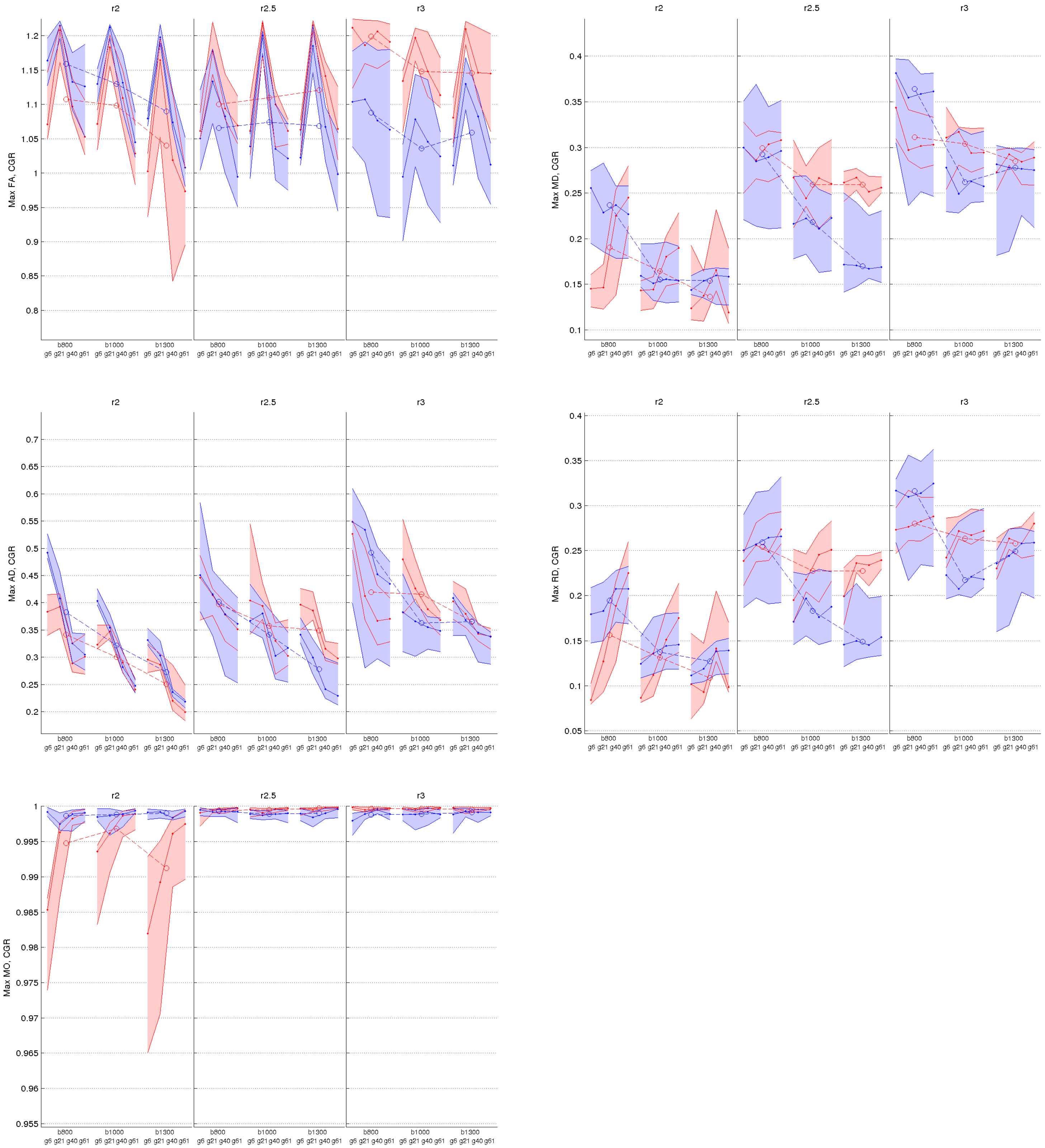

Figure 23: Maximum values for different scalar indices on the CGR.

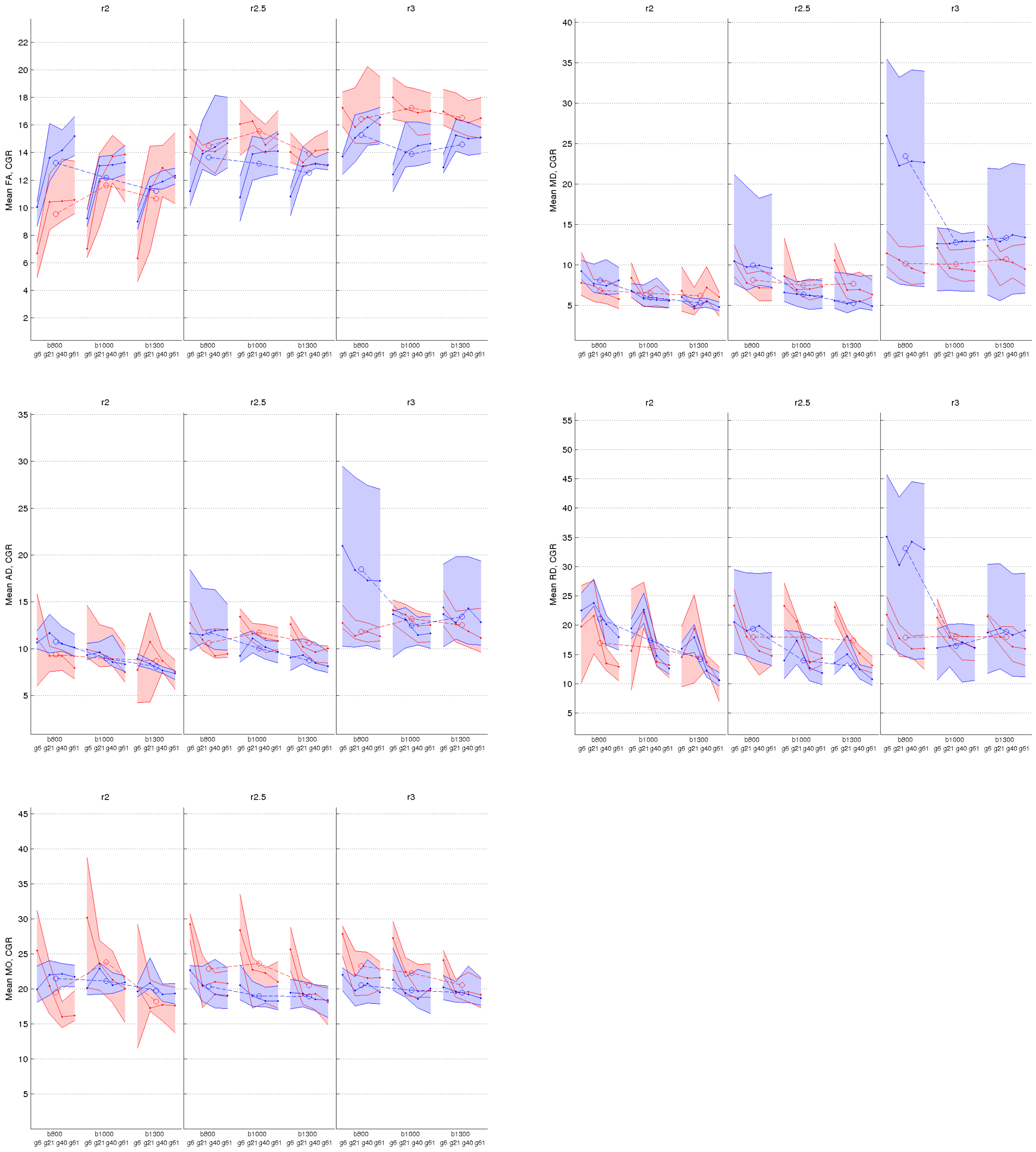

Figure 24: Coefficient of Variation values for different scalar indices on the CGR.
